# Supplementary material for: Administration of RANKL boosts thymic regeneration upon bone marrow transplantation
Source: EMBO Mol Med. 2017 Apr 28;9(6):835–51. doi: 10.15252/emmm.201607176 (PMC5452038; doi:10.15252/emmm.201607176)
Supplement: Supplementary file 1 — Appendix [file EMMM-9-835-s001.pdf]

## Appendix

# Administration of RANKL boosts thymic regeneration upon bone marrow transplantation

Noella Lopes, Hortense Vachon, Julien Marie and Magali Irla

1. Appendix figures and figure legends S1 to S9…….Page 2-10

2. Appendix table S1 ……………………………..………Page 11

3. Appendix table S2 ……………………………..…….... Page 12-22

# Appendix Figure S1

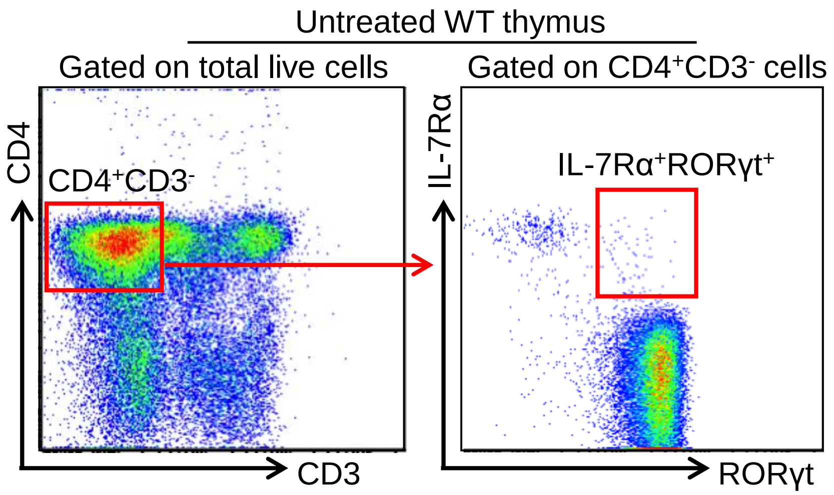


**Appendix Figure S1. Gating strategy used to identify LTi cells in the thymus.**

Thymic cells from untreated WT mice were analyzed by flow cytometry for the expression of CD4 and CD3. $CD4^{+}CD3^{-}$ cells were analyzed for the expression of IL-7Rα and RORγt. LTi cells were identified as $CD4^{+}CD3^{-}IL\text{-}7R\alpha^{+}ROR\gamma t^{+}$.

# Appendix Figure S2

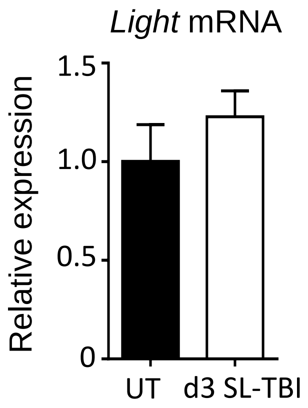


**Appendix Figure S2: LIGHT mRNA is not upregulated after SL-TBI.**

The expression of *Light* mRNA was measured by qPCR in the total thymus isolated from UT WT mice (n=4) or at d3 SL-TBI (n=4). Data information: Data are shown as mean ±SEM.

# Appendix Figure S3

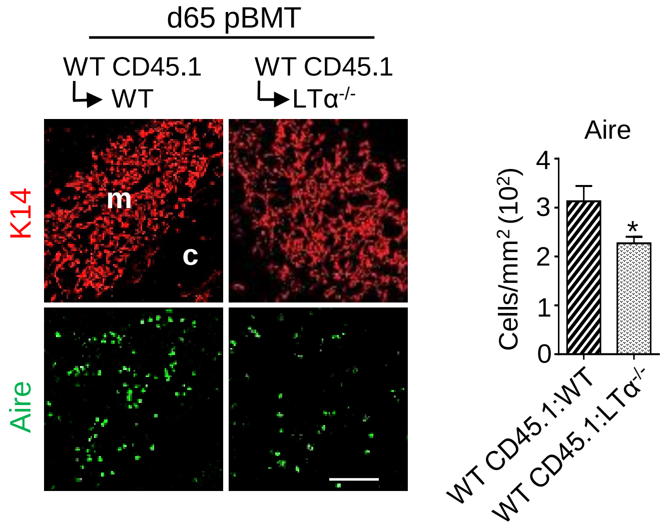


**Appendix Figure S3 : Aire$^{+}$ mTEC regeneration is altered at d65 pBMT in LTα$^{-/-}$ recipient mice.**

Thymic sections from WT CD45.1:WT and WT CD45.1:LTα$^{-/-}$ chimeras at d65 after BMT were stained for the expression of K14 and Aire. m and c denote the medulla and the cortex, respectively. The histogram shows the density of Aire$^{+}$ mTECs in medullary areas. Fifteen sections were quantified. Scale bar, 100μm.
Data information: Data are shown as mean ±SEM. Exact P-values are provided in Appendix Table S2.

# Appendix Figure S4

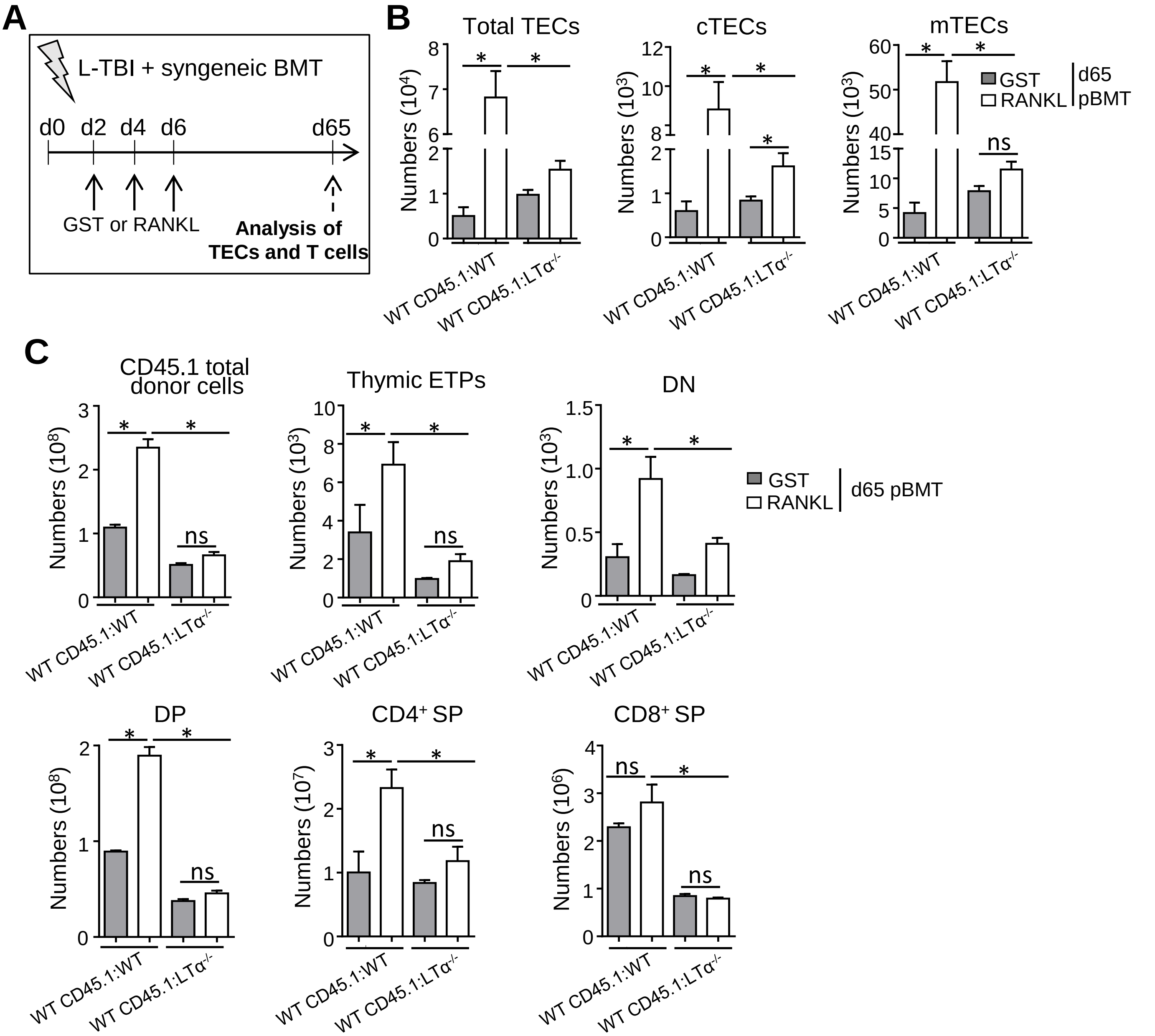


**Appendix Figure S4: RANKL administration boosts TEC regeneration and *de novo* thymopoïesis in an LTα-dependent manner during BMT.**

A Experimental setup: WT CD45.1:WT and WT CD45.1:LTα$^{-/-}$ chimeras were treated with GST or RANKL-GST proteins at d2, d4 and d6 after BMT and TEC regeneration and T-cell reconstitution were analyzed at d65 after BMT.

B-C Histograms show numbers of total TECs, cTECs and mTECs (B) and total donor cells and T-cell subsets (ETPs, DN, DP, CD4$^{+}$ SP and CD8$^{+}$ SP cells) of CD45.1 origin (C) in the thymus.

Data information: Data are shown as mean ±SEM and are pooled of 2 independent experiments with similar results (n=3 mice per group). Exact P-values are provided in Appendix Table S2.

# Appendix Figure S5

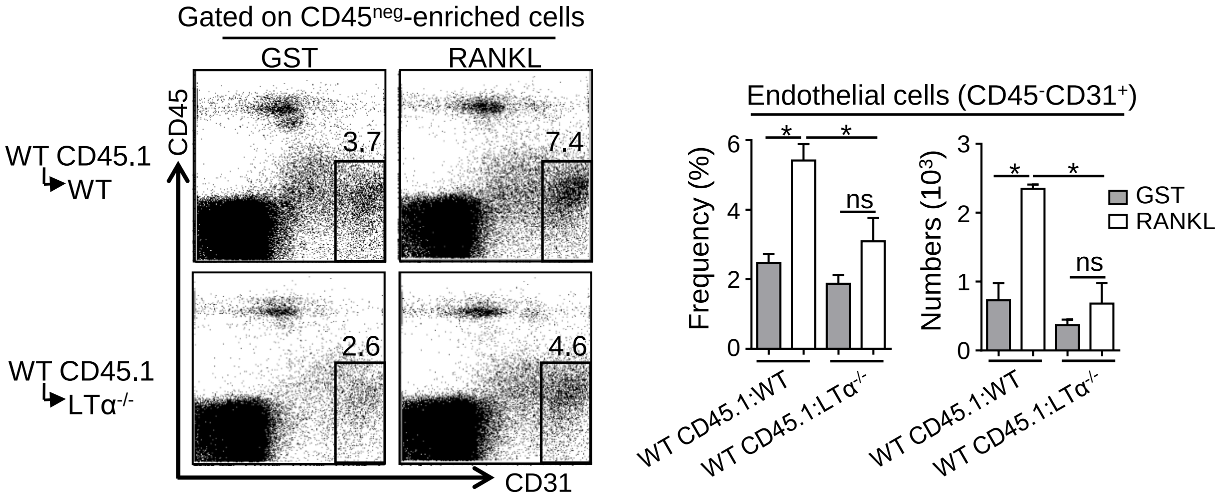


**Appendix Figure S5: RANKL treatment increases numbers of thymic endothelial cells in an LTα-dependent manner during BMT.**

Flow cytometry profiles, frequencies and numbers of $CD45^-CD31^+$ endothelial cells from the thymus of WT CD45.1:WT and WT CD45.1:LTα$^{-/-}$ chimeras treated with GST or RANKL-GST proteins at d2, d4 and d6 after BMT and analyzed at d21 after BMT.
Data are shown as mean ±SEM and are pooled of 2 independent experiments with similar results (n=3 mice per group). Exact P-values are provided in Appendix Table S2.

# Appendix Figure S6

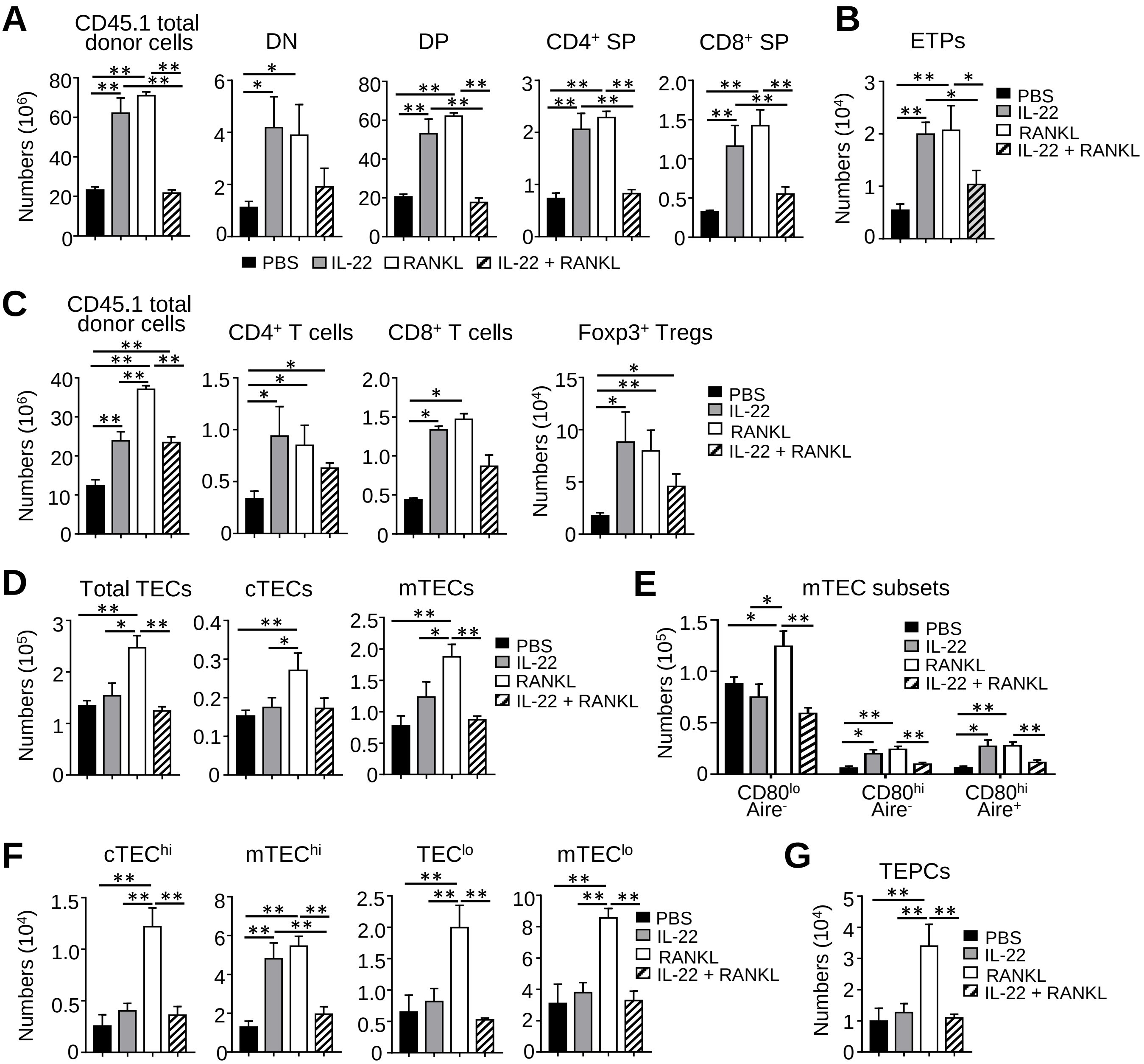


**Appendix Figure S6. The administration of RANKL and IL-22 alone boosts similarly thymopoïesis upon BMT but RANKL shows a superior capacity on TEC regeneration.**

| | |
|---|---|
| A-B | Numbers of total cells and T-cell subsets (A) and ETPs (B) of CD45.1 donor origin analyzed in the thymus. |
| C | Numbers of total donor cells, $CD4^+$ and $CD8^+$ T cells as well as $CD4^+Foxp3^+$ Tregs from CD45.1 donor origin in the spleen. |
| D,E | Numbers of total TECs, cTECs, mTECs (D) and mTEC subsets ($CD80^{lo}Aire^-$, $CD80^{hi}Aire^-$ and $CD80^{hi}Aire^+$) (E) analyzed at d21 pBMT in $CD45^{neg}$-enriched cells by AutoMACS from WT CD45.1:WT chimeras treated with PBS, IL-22, RANKL or IL-22+RANKL at d2, d4 and d6 after BMT. |
| F,G | Histograms show numbers of $cTEC^{hi}$, $mTEC^{hi}$, $TEC^{lo}$, $mTEC^{lo}$ (F) and TEPC-enriched (G). |

Data are shown as mean ±SEM and are pooled of 2 independent experiments with similar results (n=3 mice per group). Exact P-values are provided in Appendix Table S2.

# Appendix Figure S7

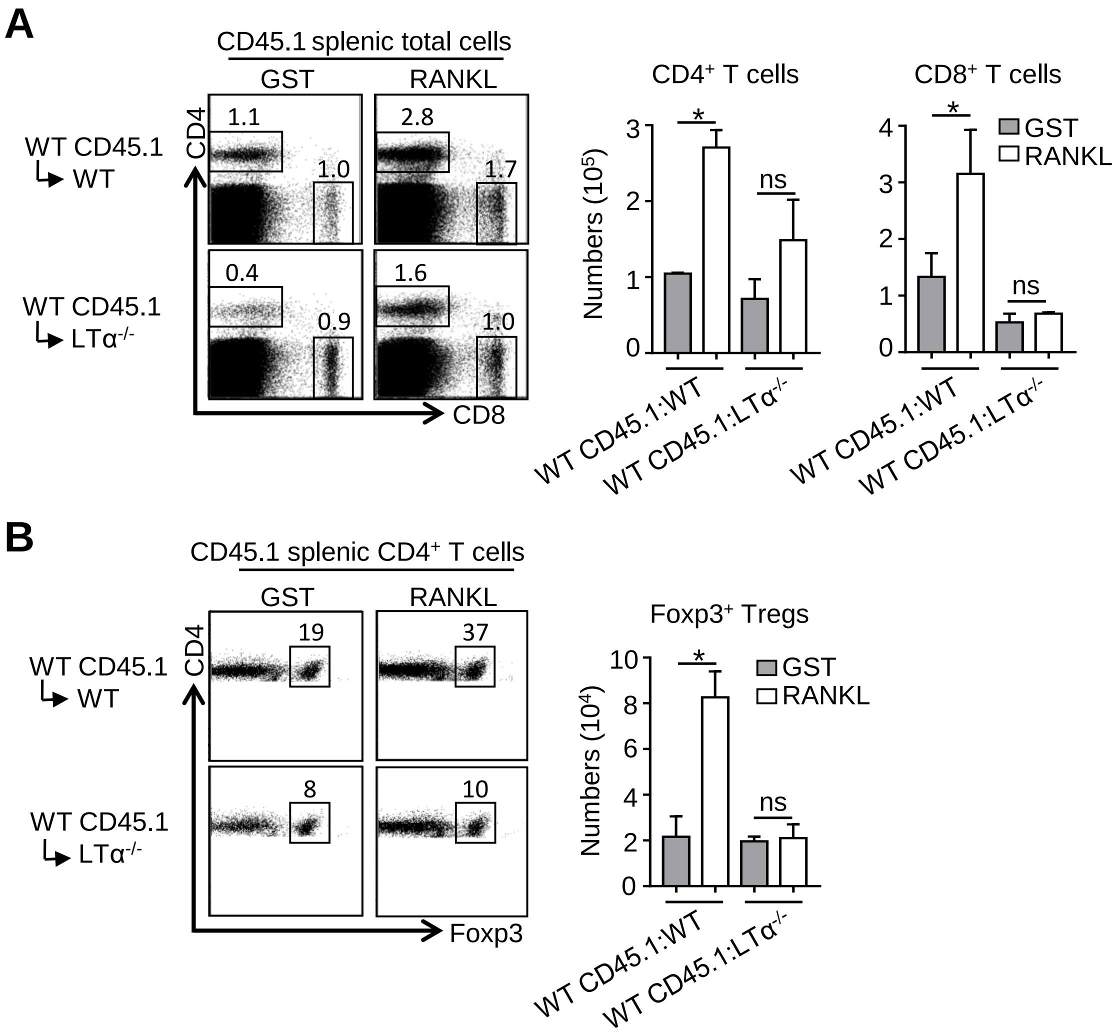


**Appendix Figure S7. Administration of RANKL protein boosts peripheral T-cell reconstitution in an LTα-dependent manner upon BMT in aged mice that show early thymic involution.**

Flow cytometry profiles and numbers of $CD4^+$ and $CD8^+$ T cells (A) as well as $CD4^+Foxp3^+$ Tregs (B) in the spleen from WT CD45.1:WT and WT CD45.1:$LT\alpha^{-/-}$ mice of 6-8 months of age treated with GST or RANKL at d2, d4 and d6 pBMT and analyzed at d21 pBMT. Significance relative to WT CD45.1:WT chimeras.
Data are shown as mean ±SEM and are pooled of 2 independent experiments with similar results (n=3 mice per group). Exact P-values are provided in Appendix Table S2.

## Appendix Figure S8

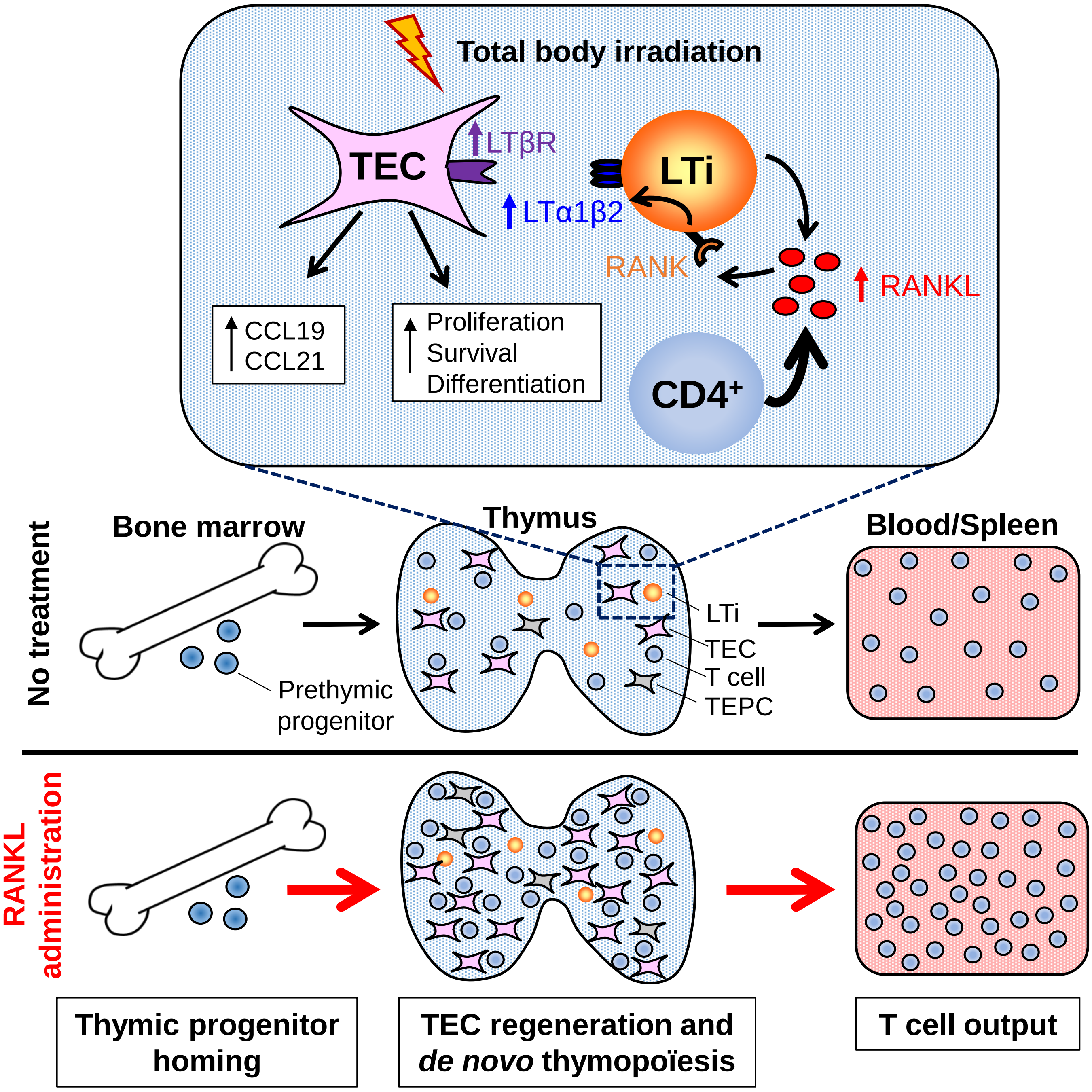


**Appendix Figure S8. RANKL administration boosts thymic regeneration upon BMT in an LTα-dependent manner.**

After total body irradiation (TBI), CD4$^+$ SP and LTi cells upregulate RANKL, which in turn increases LTα1β2 expression specifically in LTi cells. TBI also induces the upregulation of LTβR expression in all TEC subsets including TEPC-enriched cells. The expression of LTα is crucial for TEC and T-cell recovery. Importantly, the *ex vivo* administration of RANKL protein boosts TEC proliferation, survival and differentiation as well as the expansion of TEPC-enriched cells and therefore enhances TEC regeneration. Furthermore, RANKL treatment induces chemokines in TECs and adhesion molecules in endothelial cells involved in thymus homing, which ameliorates the thymic entry of T-cell progenitors, *de novo* thymopoiesis and peripheral T-cell recovery after BMT.

# Appendix Figure S9

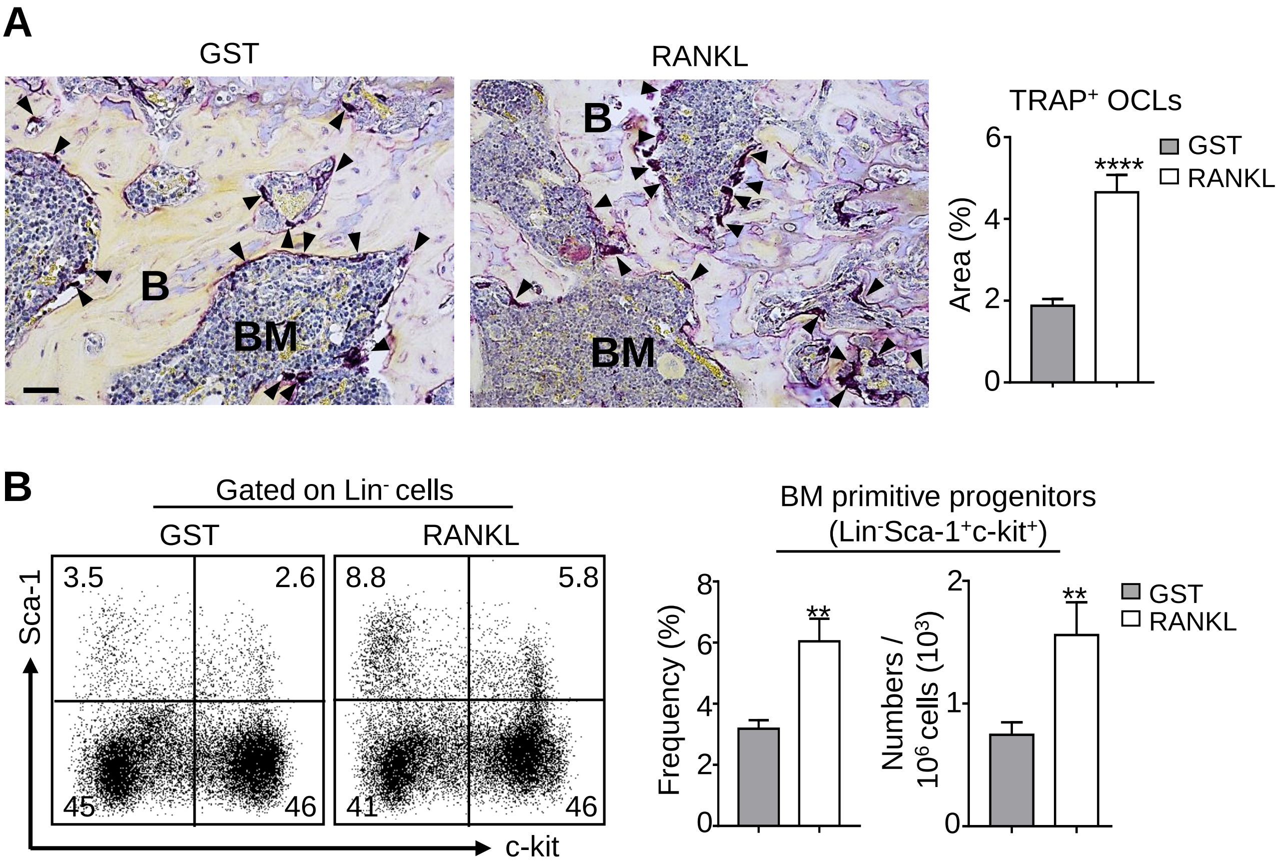


**Appendix Figure S9. RANKL administration upon BMT induces the development of TRAP$^{+}$ osteoclasts and increases primitive progenitors in the BM.**

A TRAP staining of femur epiphysis and metaphysis analyzed at d21 pBMT from WT CD45.1: WT chimeras injected with GST or RANKL at d2, d4 and d6 after BMT. Thirty five sections were quantified for each condition. Arrowheads indicate active TRAP$^{+}$ OCL stained in purple. Scale bar, 1mm. B: bone; BM: bone marrow.

B Flow cytometry profiles, percentage and numbers of BM primitive progenitors (Lin$^{-}$Sca-1$^{+}$c-kit$^{+}$) analyzed at d21 pBMT from WT CD45.1: WT chimeras injected with GST or RANKL.

Dare are shown as mean ±SEM and are pooled of 2 independent experiments with similar results (n=3 mice per group). Exact P-values are provided in Appendix Table S2.

# Appendix Table S1: Primers used for qRT-PCR

| Gene name | Forward (5'-3') | Reverse (5'-3') |
|---|---|---|
| Actin | CAGAAGGAGATTACTGCTCTGGCT | GGAGCCACCGATCCACACA |
| LTα | GCTTGGCACCCCTCCTGTC | GATGCCATGGGTCAAGTGCT |
| LIGHT | GGGCTGGTTTCTCCTGAGAC | GCTTCTCCCAGGAGCCTTTG |
| IL-22 | ACAGGTTCCAGCCCTACATG | GTCGTCACCGCTGATGTG |
| IL-23 | CCCGTATCCAGTGTGAAGATG | CCCTTTGAAGATGTCAGAGTCA |
| RANK | TGCTTGCTGCATAAAGTCTG | AGGTTTGCATTTGTCTGTGG |
| RANKL | CGCTCTGTTCCTGTACTTTC | AGAGTCGAGTCCTGCAAATC |
| CCL25 | GCCTGGTTGCCTGTTTTGTT | ACCCAGGCAGCAGTCTTCAA |
| CCL19 | GCTAATGATGCGGAAGACTG | ACTCACATCGACTCTCTAGG |
| CCL21 | GCTGCCTTAGTACAGCCAG | GTGTCTGTTCAGTTCTCTTGC |
| ICAM-1 | CCTGGTCACCGTTGTGATCC | TCAGCCACRGAGTCTCCAAG |
| VCAM-1 | TGCCGAGCTAAATTACACATTG | CCTTGTGGAGGGATGTACAGA |
| Selp | CCTAGGAACATACGGAGTCTTCACTA | CCTGAGAAATCGAATGAGACATTG |
| EpCAM | AAAGCCCCTACGACCATCAGA | TCTGATTCAGCTTATATCGAGATGTGA |
| CD31 | ACGCTGGTGCTCTATGCAAG | TCAGTTGCTGCCCATTCATCA |
| Aire | GCATAGCATCCTGGACGGCTTCC | CTGGGCTGGAGACGCTCTTTGAG |
| SP1 | TGGTGAAAATACTGGCTCTGAA | GCAGTGTTGGTATCATCAGTG |
| SP2 | TCAGACCAAAGTGGGTGACA | CTCTTGTTTCTCATTGGAGGT |
| Casein β | CTCCACTAAAGGACTTGACAG | ACCTTCTGAAGTTTCTGCTC |
| Bax | CTCAAGGCCCTGTGCACTAA | CACGGAGGAAGTCCAGTGTC |
| Bcl-xl | CGCCGGAGATAGATTTGAATAACC | CCCGGTTGCTCTGAGACATT |
| Bid | GACTCTGAGGTCAGCAACGG | CCTCCCAGTAAGCTTGCACA |
| Bak | CCAAGATCGCCTCCAGCCTA | CACGCTGGTAGACGTACAGG |
| Fezf2 | CAGCACTCTCTGCAGACACAA | TGCCGCACTGGTTACACTTA |
| Apoc3 | CCTCTTGGCTCTCCTGGCATCT | TGCTCCAGTAGCCTTTCAGGG |
| Fabp9 | CACTGCAGACAACCGAAAAG | TCTGTTTGCCAAGCCATTTT |
| Resp18 | TCAGTCAGCAACAAGGTTGAGGCCCAC | CCAGCCAAGATGCAGAGTTCGTTAAAG |

**Appendix Table S2: Exact p-values derived from statistical tests.**

| Figure | Comparison | p-values | Category | Test |
|---|---|---|---|---|
| Figure 1A | CD45+ cells; UT vs d3 SL-TBI | <0.0001 | **** | T-test |
| Figure 1B | DP; UT vs d3 SL-TBI | <0.0001 | **** | T-test |
| | CD4+ SP; UT vs d3 SL-TBI | <0.0001 | **** | T-test |
| | CD8+ SP; UT vs d3 SL-TBI | <0.0001 | **** | T-test |
| Figure 1C | LTi; UT vs d3 SL-TBI | 0.009 | ** | T-test |
| Figure 1D | CD4+ SP; UT vs d3 SL-TBI | 0.0500 | * | Mann Whitney test, two tailed |
| | LTi ; UT vs d3 SL-TBI | 0.0143 | * | Mann Whitney test, two tailed |
| | LTi ; UT vs d3 L-TBI | 0.0143 | * | Mann Whitney test, two tailed |
| Figure 1E | WT UT vs WT d3 SL-TBI | 0.003 | *** | Mann Whitney test, one tailed |
| | WT d3 SL-TBI vs Rag2-/- d3 SL-TBI | 0.0159 | * | Mann Whitney test, one tailed |
| | Rag2-/- d3 SL-TBI vs Rag2-/- d3 SL-TBI | 0.0368 | * | Mann Whitney test, one tailed |
| Figure 1F | LTi ; d0 vs d3 | 0.0006 | *** | Mann Whitney test, two tailed |
| | LTi ; d0 vs d6 | 0.0003 | *** | Mann Whitney test, two tailed |
| | LTi ; d0 vs d10 | 0.0003 | *** | Mann Whitney test, two tailed |
| | CD4+; d0 vs d3 | 0.0006 | *** | Mann Whitney test, two tailed |
| | CD4+ ; d0 vs d6 | 0.0003 | *** | Mann Whitney test, two tailed |
| | CD4+; d0 vs d10 | 0.0197 | * | Mann Whitney test, two tailed |
| | CD4+ SP vs LTi post-SL-TBI | 0.0417 | * | nonparametric Spearman correlation test |
| Figure 2A | Total TECs; UT vs PBS | <0.0001 | **** | Mann Whitney test, two tailed |
| | Total TECs; GST vs RANKL | 0.0286 | * | Mann Whitney test, two tailed |
| | Total TECs; isotype vs IK22/5 | 0.0286 | * | Mann Whitney test, two tailed |
| | Total TECs; PBS vs IK22/5 | 0.0002 | *** | Mann Whitney test, two tailed |
| | Total TECs; RANKL vs IK22/5 | 0.095 | * | Mann Whitney test, two tailed |
| | cTECs; UT vs PBS | 0.0007 | *** | Mann Whitney test, two tailed |
| | cTECs; GST vs RANKL | 0.0238 | * | Mann Whitney test, two tailed |
| | cTECs; isotype vs IK22/5 | 0.0087 | ** | Mann Whitney test, two tailed |
| | cTECs; PBS vs IK22/5 | 0.0007 | *** | Mann Whitney test, two tailed |
| | cTECs; RANKL vs IK22/5 | 0.0238 | * | Mann Whitney test, two tailed |
| | mTECs; UT vs PBS | 0.0007 | *** | Mann Whitney test, two tailed |
| | mTECs; GST vs RANKL | 0.0500 | * | Mann Whitney test, two tailed |
| | mTECs; isotype vs IK22/5 | 0.0238 | * | Mann Whitney test, two tailed |
| | mTECs; PBS vs IK22/5 | 0.0007 | *** | Mann Whitney test, two tailed |
| | mTECs; RANKL vs IK22/5 | 0.0238 | * | Mann Whitney test, two tailed |
| | CD80loAire-; UT vs PBS | 0.0007 | *** | Mann Whitney test, two tailed |
| | CD80hiAire-; UT vs PBS | 0.0004 | *** | Mann Whitney test, two tailed |
| | CD80hiAire-; GST vs RANKL | 0.0167 | * | Mann Whitney test, two tailed |
| | CD80hiAire-; isotype vs IK22/5 | 0.0238 | * | Mann Whitney test, two tailed |
| | CD80hiAire-; PBS vs IK22/5 | 0.0004 | *** | Mann Whitney test, two tailed |
| | CD80hiAire-; RANKL vs IK22/5 | 0.0012 | ** | Mann Whitney test, two tailed |
| | CD80hiAire+; UT vs PBS | 0.0004 | *** | Mann Whitney test, two tailed |
| | CD80hiAire+; GST vs RANKL | 0.0079 | ** | Mann Whitney test, two tailed |
| | CD80hiAire+; isotype vs IK22/5 | 0.0238 | * | Mann Whitney test, two tailed |
| | CD80hiAire+; PBS vs IK22/5 | 0.0004 | *** | Mann Whitney test, two tailed |
| | CD80hiAire+; RANKL vs IK22/5 | 0.0043 | ** | Mann Whitney test, two tailed |

| | | | | |
|---|---|---|---|---|
| Figure 2B | cTEChi; UT vs PBS | 0.0047 | ** | Mann Whitney test, two tailed |
| | cTEChi; GST vs RANKL | 0.0500 | * | Mann Whitney test, two tailed |
| | cTEChi; isotype vs IK22/5 | 0.0238 | * | Mann Whitney test, two tailed |
| | cTEChi; PBS vs IK22/5 | 0.0007 | *** | Mann Whitney test, two tailed |
| | cTEChi; RANKL vs IK22/5 | 0.0238 | * | Mann Whitney test, two tailed |
| | mTEChi; UT vs PBS | 0.0007 | *** | Mann Whitney test, two tailed |
| | mTEChi; GST vs RANKL | 0.0500 | * | Mann Whitney test, two tailed |
| | mTEChi; isotype vs IK22/5 | 0.0476 | * | Mann Whitney test, two tailed |
| | mTEChi; PBS vs IK22/5 | 0.0007 | *** | Mann Whitney test, two tailed |
| | mTEChi; RANKL vs IK22/5 | 0.0238 | * | Mann Whitney test, two tailed |
| | TEClo; UT vs PBS | 0.0007 | *** | Mann Whitney test, two tailed |
| | TEClo; GST vs RANKL | 0.0500 | * | Mann Whitney test, two tailed |
| | TEClo; PBS vs IK22/5 | 0.0047 | ** | Mann Whitney test, two tailed |
| | TEClo; RANKL vs IK22/5 | 0.0238 | * | Mann Whitney test, two tailed |
| | mTEClo; UT vs PBS | 0.0007 | *** | Mann Whitney test, two tailed |
| | mTEClo; GST vs RANKL | 0.0500 | * | Mann Whitney test, two tailed |
| | mTEClo; isotype vs IK22/5 | 0.0238 | * | Mann Whitney test, two tailed |
| | mTEClo; PBS vs IK22/5 | 0.0080 | ** | Mann Whitney test, two tailed |
| | mTEClo; RANKL vs IK22/5 | 0.0238 | * | Mann Whitney test, two tailed |
| Figure 2C | TEPC-enriched cells; UT vs PBS | 0.0093 | ** | Mann Whitney test, two tailed |
| | TEPC-enriched cells; GST vs RANKL | 0.0500 | * | Mann Whitney test, two tailed |
| | TEPC-enriched cells; isotype vs IK22/5 | 0.0357 | * | Mann Whitney test, two tailed |
| | TEPC-enriched cells; PBS vs IK22/5 | 0.0016 | ** | Mann Whitney test, two tailed |
| | TEPC-enriched cells; RANKL vs IK22/5 | 0.0357 | * | Mann Whitney test, two tailed |
| Figure 2D | Ki-67+ cTECs; UT vs PBS | 0.0007 | *** | Mann Whitney test, two tailed |
| | Ki-67+ cTECs; GST vs RANKL | 0.0500 | * | Mann Whitney test, two tailed |
| | Ki-67+ cTECs; isotype vs IK22/5 | 0.0022 | ** | Mann Whitney test, two tailed |
| | Ki-67+ cTECs; PBS vs IK22/5 | 0.0007 | *** | Mann Whitney test, two tailed |
| | Ki-67+ cTECs; RANKL vs IK22/5 | 0.0238 | * | Mann Whitney test, two tailed |
| | Ki-67+ mTECs; UT vs PBS | 0.0007 | *** | Mann Whitney test, two tailed |
| | Ki-67+ mTECs; GST vs RANKL | 0.0500 | * | Mann Whitney test, two tailed |
| | Ki-67+ mTECs; isotype vs IK22/5 | 0.0022 | ** | Mann Whitney test, two tailed |
| | Ki-67+ mTECs; PBS vs IK22/5 | 0.0007 | *** | Mann Whitney test, two tailed |
| | Ki-67+ mTECs; RANKL vs IK22/5 | 0.0022 | ** | Mann Whitney test, two tailed |
| | Ki-67+ TEPC-enriched cells; UT vs PBS | 0.0127 | * | Mann Whitney test, two tailed |
| | Ki-67+ TEPC-enriched cells; GST vs RANKL | 0.0500 | * | Mann Whitney test, two tailed |
| | Ki-67+ TEPC-enriched cells; isotype vs IK22/5 | 0.0238 | * | Mann Whitney test, two tailed |
| | Ki-67+ TEPC-enriched cells; PBS vs IK22/5 | 0.0077 | ** | Mann Whitney test, two tailed |
| | Ki-67+ TEPC-enriched cells; RANKL vs IK22/5 | 0.0238 | * | Mann Whitney test, two tailed |
| Figure 2E | Bax; cTECs; GST vs RANKL | 0.0500 | * | Mann Whitney test, one tailed |
| | Bcl-xl; cTECs; GST vs RANKL | 0.0500 | * | Mann Whitney test, one tailed |
| | Bax; mTECs; GST vs RANKL | 0.0500 | * | Mann Whitney test, one tailed |
| | Bid; mTECs; GST vs RANKL | 0.0500 | * | Mann Whitney test, one tailed |
| | Bad; mTECs; GST vs RANKL | 0.0500 | * | Mann Whitney test, one tailed |
| Figure 2F | Aire; GST vs RANKL | 0.0004 | *** | T-test |
| Figure 2G | Aire; GST vs RANKL | 0.0500 | * | Mann Whitney test, one tailed |
| | SP1; GST vs RANKL | 0.0500 | * | Mann Whitney test, one tailed |
| | SP2; GST vs RANKL | 0.0500 | * | Mann Whitney test, one tailed |
| Figure 2H | Selp; GST vs RANKL | 0.0500 | * | Mann Whitney test, one tailed |

| | | | | |
|---|---|---|---|---|
| | ICAM-1; GST vs RANKL | 0.0500 | * | Mann Whitney test, one tailed |
| | CCL21; GST vs RANKL | 0.0500 | * | Mann Whitney test, one tailed |
| Figure 3A | UT vs SL-TBI | 0.0040 | ** | Mann Whitney test, two tailed |
| Figure 3B | GST vs RANKL | 0.0286 | * | Mann Whitney test, one tailed |
| Figure 3C | LTα protein expression in hematopoietic cells after RANKL *in vivo* stimulation | <0.0001 | **** | One-Way ANOVA |
| Figure 3D | UT vs PBS | 0.0022 | ** | Mann Whitney test, one tailed |
| | Isotype vs IK22/5 | 0.0500 | * | Mann Whitney test, one tailed |
| Figure 3E | LTα protein expression in hematopoietic cells after RANKL *in vivo* neutralization | 0.003 | *** | One-Way ANOVA |
| Figure 3F | LTα mRNA; WT UT vs WT d3 SL-TBI | 0.0011 | ** | Mann Whitney test, two tailed |
| | LTα mRNA; ZAP-70-/- UT vs ZAP-70-/- d3 SL-TBI | 0.0011 | ** | Mann Whitney test, two tailed |
| Figure 3H | RANKL vs LTα during the course of pBMT | 0.0100 | * | nonparametric Spearman correlation test |
| Figure 3I | LTα protein; UT vs d3 | 0.0095 | ** | Mann Whitney test, two tailed |
| | LTα protein; UT vs d6 | 0.0095 | ** | Mann Whitney test, two tailed |
| Figure 3J | LTα protein; d3; donor vs host | <0.0001 | **** | T-test |
| | LTα protein; d6; donor vs host | <0.0001 | **** | T-test |
| Figure 3L | LTα mRNA; UT vs d3 SL-TBI | 0.0036 | ** | Mann Whitney test, one tailed |
| | LTβ mRNA; UT vs d3 SL-TBI | 0.0286 | * | Mann Whitney test, one tailed |
| Figure 3M | CD45+; UT vs d3 SL-TBI | 0.0022 | ** | Mann Whitney test, one tailed |
| Figure 3N | LTα protein; UT vs d3 SL-TBI | 0.0022 | ** | Mann Whitney test, two tailed |
| | LTα protein; d3 SL-TBI vs d3 L-TBI | 0.0022 | ** | Mann Whitney test, two tailed |
| Figure 3O | LTβR-Fc; UT vs d3 SL-TBI | 0.0500 | * | Mann Whitney test, one tailed |
| | LTβR-Fc; d3 SL-TBI vs d3 L-TBI | 0.0500 | * | Mann Whitney test, one tailed |
| Figure 4A | TECs; UT vs d3 SL-TBI | 0.0286 | * | Mann Whitney test, two tailed |
| | cTECs; UT vs d3 SL-TBI | 0.0286 | * | Mann Whitney test, two tailed |
| | mTECs; UT vs d3 SL-TBI | 0.0286 | * | Mann Whitney test, two tailed |
| | TEPCs; UT vs d3 SL-TBI | 0.0286 | * | Mann Whitney test, two tailed |
| Figure 4B | D10; WT CD45.1:WT vs WT CD45.1: LTα-/- | 0.0143 | * | Mann Whitney test, one tailed |
| | D21; WT CD45.1:WT vs WT CD45.1: LTα-/- | 0.0143 | * | Mann Whitney test, one tailed |
| | D65; WT CD45.1:WT vs WT CD45.1: LTα-/- | 0.0040 | ** | Mann Whitney test, one tailed |
| Figure 4C | cTECs; D21; WT CD45.1:WT vs WT CD45.1: LTα-/- | 0.0143 | * | Mann Whitney test, one tailed |
| | cTECs; D65; WT CD45.1:WT vs WT CD45.1: LTα-/- | 0.0040 | ** | Mann Whitney test, one tailed |
| | mTECs; D10; WT CD45.1:WT vs WT CD45.1: LTα-/- | 0.0143 | * | Mann Whitney test, one tailed |
| | mTECs; D21; WT CD45.1:WT vs WT CD45.1: LTα-/- | 0.0286 | * | Mann Whitney test, one tailed |
| | mTECs; D65; WT CD45.1:WT vs WT CD45.1: LTα-/- | 0.0079 | ** | Mann Whitney test, one tailed |
| Figure 4D | CD80loAire- mTECs; D10; WT CD45.1:WT vs WT CD45.1: LTα-/- | 0.0286 | * | Mann Whitney test, one tailed |
| | CD80loAire- mTECs; D21; WT CD45.1:WT vs WT CD45.1: LTα-/- | 0.0143 | * | Mann Whitney test, one tailed |
| | CD80loAire- mTECs; D65; WT CD45.1:WT vs WT CD45.1: LTα-/- | 0.0040 | ** | Mann Whitney test, one tailed |
| | CD80hiAire- mTECs; D10; WT CD45.1:WT vs WT CD45.1: LTα-/- | 0.0286 | * | Mann Whitney test, one tailed |
| | CD80hiAire- mTECs; D21; WT CD45.1:WT vs WT CD45.1: LTα-/- | 0.0143 | * | Mann Whitney test, one tailed |
| | CD80hiAire- mTECs; D65; WT CD45.1:WT vs WT CD45.1: LTα-/- | 0.0079 | ** | Mann Whitney test, one tailed |

| | | | | |
|---|---|---|---|---|
| | CD80hiAire+ mTECs; D10; WT CD45.1:WT vs WT CD45.1: LTα-/- | 0.0143 | * | Mann Whitney test, one tailed |
| | CD80hiAire+ mTECs; D21; WT CD45.1:WT vs WT CD45.1: LTα-/- | 0.0143 | * | Mann Whitney test, one tailed |
| | CD80hiAire+ mTECs; D65; WT CD45.1:WT vs WT CD45.1: LTα-/- | 0.0040 | ** | Mann Whitney test, one tailed |
| Figure 4E | cTEChi; D21; WT CD45.1:WT vs WT CD45.1: LTα-/- | 0.0500 | * | Mann Whitney test, one tailed |
| | cTEChi; D65; WT CD45.1:WT vs WT CD45.1: LTα-/- | 0.0143 | * | Mann Whitney test, one tailed |
| | mTEChi; D10; WT CD45.1:WT vs WT CD45.1: LTα-/- | 0.0143 | * | Mann Whitney test, one tailed |
| | mTEChi; D21; WT CD45.1:WT vs WT CD45.1: LTα-/- | 0.0500 | * | Mann Whitney test, one tailed |
| | mTEChi; D65; WT CD45.1:WT vs WT CD45.1: LTα-/- | 0.0179 | * | Mann Whitney test, one tailed |
| | TEClo; D10; WT CD45.1:WT vs WT CD45.1: LTα-/- | 0.0143 | * | Mann Whitney test, one tailed |
| | TEClo; D21; WT CD45.1:WT vs WT CD45.1: LTα-/- | 0.0500 | * | Mann Whitney test, one tailed |
| | TEClo; D65; WT CD45.1:WT vs WT CD45.1: LTα-/- | 0.0286 | * | Mann Whitney test, one tailed |
| | mTEClo; D10; WT CD45.1:WT vs WT CD45.1: LTα-/- | 0.0143 | * | Mann Whitney test, one tailed |
| | mTEClo; D21; WT CD45.1:WT vs WT CD45.1: LTα-/- | 0.0500 | * | Mann Whitney test, one tailed |
| | mTEClo; D65; WT CD45.1:WT vs WT CD45.1: LTα-/- | 0.0079 | ** | Mann Whitney test, one tailed |
| Figure 4F | TEPC-enriched cells; D10; WT CD45.1:WT vs WT CD45.1: LTα-/- | 0.0143 | * | Mann Whitney test, one tailed |
| | TEPC-enriched cells; D21; WT CD45.1:WT vs WT CD45.1: LTα-/- | 0.0079 | ** | Mann Whitney test, one tailed |
| | TEPC-enriched cells; D65; WT CD45.1:WT vs WT CD45.1: LTα-/- | 0.0286 | * | Mann Whitney test, one tailed |
| Figure 4G | Total TECs; D21; WT CD45.1:WT vs WT CD45.1: LTα-/- | 0.0286 | * | Mann Whitney test, one tailed |
| | Total TECs; D65; WT CD45.1:WT vs WT CD45.1: LTα-/- | 0.0040 | ** | Mann Whitney test, one tailed |
| | cTECs; D10; WT CD45.1:WT vs WT CD45.1: LTα-/- | 0.0500 | * | Mann Whitney test, one tailed |
| | cTECs; D21; WT CD45.1:WT vs WT CD45.1: LTα-/- | 0.0143 | * | Mann Whitney test, one tailed |
| | cTECs; D65; WT CD45.1:WT vs WT CD45.1: LTα-/- | 0.0040 | ** | Mann Whitney test, one tailed |
| | mTECs; D10; WT CD45.1:WT vs WT CD45.1: LTα-/- | 0.0143 | * | Mann Whitney test, one tailed |
| | mTECs; D21; WT CD45.1:WT vs WT CD45.1: LTα-/- | 0.0143 | * | Mann Whitney test, one tailed |
| | mTECs; D65; WT CD45.1:WT vs WT CD45.1: LTα-/- | 0.0040 | ** | Mann Whitney test, one tailed |
| | TEPC-enriched cells; D10; WT CD45.1:WT vs WT CD45.1: LTα-/- | 0.0286 | * | Mann Whitney test, one tailed |
| | TEPC-enriched cells; D21; WT CD45.1:WT vs WT CD45.1: LTα-/- | 0.0079 | ** | Mann Whitney test, one tailed |
| | TEPC-enriched cells; D65; WT CD45.1:WT vs WT CD45.1: LTα-/- | 0.0286 | * | Mann Whitney test, one tailed |
| Figure 4H | Aire; WT CD45.1:WT vs WT CD45.1: LTα-/- | 0.0500 | * | Mann Whitney test, one tailed |
| | SP1; WT CD45.1:WT vs WT CD45.1: LTα-/- | 0.0286 | * | Mann Whitney test, one tailed |
| | SP2; WT CD45.1:WT vs WT CD45.1: LTα-/- | 0.0143 | * | Mann Whitney test, one tailed |
| | Casein β; WT CD45.1:WT vs WT CD45.1: LTα-/- | 0.0143 | * | Mann Whitney test, one tailed |
| | Fezf2; WT CD45.1:WT vs WT CD45.1: LTα-/- | 0.0143 | * | Mann Whitney test, one tailed |
| | Apoc3; WT CD45.1:WT vs WT CD45.1: LTα-/- | 0.0143 | * | Mann Whitney test, one tailed |
| | Fabp9; WT CD45.1:WT vs WT CD45.1: LTα-/- | 0.0143 | * | Mann Whitney test, one tailed |
| | Resp18; WT CD45.1:WT vs WT CD45.1: LTα-/- | 0.0143 | * | Mann Whitney test, one tailed |
| Figure 5A | CD45.1 total donor cells; D10; WT CD45.1:WT vs WT CD45.1: LTα-/- | 0.0061 | ** | Mann Whitney test, one tailed |

| | | | | |
|---|---|---|---|---|
| | CD45.1 total donor cells; D21; WT CD45.1:WT vs WT CD45.1: LTα-/- | 0.0006 | *** | Mann Whitney test, one tailed |
| | CD45.1 total donor cells; D65; WT CD45.1:WT vs WT CD45.1: LTα-/- | 0.0010 | *** | Mann Whitney test, one tailed |
| | DN; D10; WT CD45.1:WT vs WT CD45.1: LTα-/- | 0.0500 | * | Mann Whitney test, one tailed |
| | DN; D21; WT CD45.1:WT vs WT CD45.1: LTα-/- | 0.0143 | * | Mann Whitney test, one tailed |
| | DN; D65; WT CD45.1:WT vs WT CD45.1: LTα-/- | 0.0486 | * | Mann Whitney test, one tailed |
| | DP; D10; WT CD45.1:WT vs WT CD45.1: LTα-/- | 0.0384 | * | Mann Whitney test, one tailed |
| | DP; D21; WT CD45.1:WT vs WT CD45.1: LTα-/- | 0.0014 | ** | Mann Whitney test, one tailed |
| | DP; D65; WT CD45.1:WT vs WT CD45.1: LTα-/- | 0.0023 | ** | Mann Whitney test, one tailed |
| | CD4+ SP; D10; WT CD45.1:WT vs WT CD45.1: LTα-/- | 0.0500 | * | Mann Whitney test, one tailed |
| | CD4+ SP; D21; WT CD45.1:WT vs WT CD45.1: LTα-/- | 0.0143 | * | Mann Whitney test, one tailed |
| | CD4+ SP; D65; WT CD45.1:WT vs WT CD45.1: LTα-/- | 0.0079 | ** | Mann Whitney test, one tailed |
| | CD8+ SP; D21; WT CD45.1:WT vs WT CD45.1: LTα-/- | 0.0143 | * | Mann Whitney test, one tailed |
| | CD8+ SP; D65; WT CD45.1:WT vs WT CD45.1: LTα-/- | 0.0079 | ** | Mann Whitney test, one tailed |
| Figure 5B | DN1; D10; WT CD45.1:WT vs WT CD45.1: LTα-/- | 0.0500 | * | Mann Whitney test, one tailed |
| | DN1; D21; WT CD45.1:WT vs WT CD45.1: LTα-/- | 0.0143 | * | Mann Whitney test, one tailed |
| | DN2; D10; WT CD45.1:WT vs WT CD45.1: LTα-/- | 0.0500 | * | Mann Whitney test, one tailed |
| | DN2; D21; WT CD45.1:WT vs WT CD45.1: LTα-/- | 0.0179 | * | Mann Whitney test, one tailed |
| | DN3; D10; WT CD45.1:WT vs WT CD45.1: LTα-/- | 0.0500 | * | Mann Whitney test, one tailed |
| | DN3; D21; WT CD45.1:WT vs WT CD45.1: LTα-/- | 0.0286 | * | Mann Whitney test, one tailed |
| | DN4; D10; WT CD45.1:WT vs WT CD45.1: LTα-/- | 0.0500 | * | Mann Whitney test, one tailed |
| | DN4; D21; WT CD45.1:WT vs WT CD45.1: LTα-/- | 0.0143 | * | Mann Whitney test, one tailed |
| Figure 5C | Thymic ETPs; WT CD45.1:WT vs WT CD45.1: LTα-/- | 0.0143 | * | Mann Whitney test, one tailed |
| Figure 5D | CCL19; TECs; WT CD45.1:WT vs WT CD45.1: LTα-/- | 0.0008 | *** | T-test |
| | CCL19; Fibroblasts; WT CD45.1:WT vs WT CD45.1: LTα-/- | 0.0015 | ** | T-test |
| | CCL21; TECs; WT CD45.1:WT vs WT CD45.1: LTα-/- | 0.0023 | ** | T-test |
| | CCL21; Fibroblasts; WT CD45.1:WT vs WT CD45.1: LTα-/- | 0.0396 | * | T-test |
| | ICAM-1; Endothelial cells; WT CD45.1:WT vs WT CD45.1: LTα-/- | 0.0394 | * | T-test |
| | VCAM-1; Endothelial cells; WT CD45.1:WT vs WT CD45.1: LTα-/- | 0.0011 | ** | T-test |
| | Selp; Endothelial cells; WT CD45.1:WT vs WT CD45.1: LTα-/- | 0.0092 | ** | T-test |
| Figure 5E | CD45.1 ETPs; WT CD45.1:WT vs WT CD45.1: LTα-/- | 0.0011 | ** | Mann Whitney test, one tailed |
| | CD45.1 donor cells; WT CD45.1:WT vs WT CD45.1: LTα-/- | 0.0011 | ** | Mann Whitney test, one tailed |
| Figure 5F | IL-22; WT; UT vs d3 SL-TBI | 0.0362 | * | T-test |
| | IL-22; LTα-/-; UT vs d3 SL-TBI | 0.0096 | ** | T-test |
| Figure 5G | IL-23; total thymus; WT; UT vs d3 SL-TBI | 0.0117 | * | T-test |
| | IL-23; total thymus; LTα-/-; UT vs d3 SL-TBI | 0.0172 | * | T-test |
| | IL-23; purified DCs; WT; UT vs d3 SL-TBI | 0.0011 | ** | T-test |
| | IL-23; purified DCs; LTα-/-; UT vs d3 SL-TBI | 0.0184 | * | T-test |
| Figure 6B | GST vs RANKL-GST | 0.0184 | * | T-test |
| Figure 6C | WT CD45.1:WT GST vs WT CD45.1:WT RANKL | 0.0178 | * | T-test |
| Figure 6D | Total TECs; WT CD45.1:WT GST vs WT CD45.1: WT RANKL | 0.0003 | *** | T-test |

| | | | | |
|---|---|---|---|---|
| | Total TECs; WT CD45.1:LTα-/- GST vs WT CD45.1: LTα-/- RANKL | 0.0002 | *** | T-test |
| | cTECs; WT CD45.1:WT GST vs WT CD45.1:WT RANKL | 0.0002 | *** | T-test |
| | cTECs; WT CD45.1:WT RANKL vs WT CD45.1: LTα-/- RANKL | 0.0015 | ** | T-test |
| | cTECs; WT CD45.1:LTα-/- GST vs WT CD45.1: LTα-/- RANKL | <0.0001 | **** | T-test |
| | mTECs; WT CD45.1:WT GST vs WT CD45.1:WT RANKL | <0.0001 | **** | T-test |
| | mTECs; WT CD45.1:LTα-/- GST vs WT CD45.1: LTα-/- RANKL | 0.0007 | *** | T-test |
| Figure 6E | WT CD45.1:WT GST vs WT CD45.1:WT RANKL | 0.0007 | *** | T-test |
| | WT CD45.1:WT RANKL vs WT CD45.1: LTα-/- RANKL | <0.0001 | **** | T-test |
| Figure 6F | SP1; WT CD45.1:WT GST vs WT CD45.1:WT RANKL | 0.0015 | ** | T-test |
| | SP1; WT CD45.1:WT RANKL vs WT CD45.1: LTα-/- RANKL | 0.0315 | * | T-test |
| | SP2; WT CD45.1:WT GST vs WT CD45.1:WT RANKL | 0.0315 | * | T-test |
| | SP2; WT CD45.1:WT RANKL vs WT CD45.1: LTα-/- RANKL | 0.0140 | * | T-test |
| Figure 6G | CD45.1 total cells - WT CD45.1:WT GST vs WT CD45.1:WT RANKL | 0.0003 | *** | T-test |
| | CD45.1 total cells - WT CD45.1:WT RANKL vs WT CD45.1: LTα-/- RANKL | 0.0014 | ** | T-test |
| | CD45.1 total cells - WT CD45.1: LTα-/- GST vs WT CD45.1: LTα-/- RANKL | 0.0260 | * | T-test |
| | Thymic ETPs - WT CD45.1:WT GST vs WT CD45.1:WT RANKL | 0.0004 | *** | T-test |
| | Thymic ETPs - WT CD45.1:WT RANKL vs WT CD45.1: LTα-/- RANKL | 0.0367 | * | T-test |
| | Thymic ETPs - WT CD45.1:LTα-/- GST vs WT CD45.1: LTα-/- RANKL | 0.0312 | * | T-test |
| | DN - WT CD45.1:WT GST vs WT CD45.1:WT RANKL | 0.0495 | * | T-test |
| | DN - WT CD45.1:WT RANKL vs WT CD45.1: LTα-/- RANKL | 0.0443 | * | T-test |
| | DP - WT CD45.1:WT GST vs WT CD45.1:WT RANKL | 0.0040 | ** | T-test |
| | DP - WT CD45.1:WT RANKL vs WT CD45.1: LTα-/- RANKL | 0.0009 | *** | T-test |
| | CD4+ SP - WT CD45.1:WT GST vs WT CD45.1:WT RANKL | 0.0005 | *** | T-test |
| | CD4+ SP - WT CD45.1:WT RANKL vs WT CD45.1: LTα-/- RANKL | 0.0003 | *** | T-test |
| | CD4+ SP - WT CD45.1: LTα-/- GST vs WT CD45.1: LTα-/- RANKL | 0.0346 | * | T-test |
| | CD8+ SP - WT CD45.1:WT GST vs WT CD45.1:WT RANKL | 0.0185 | * | T-test |
| | CD8+ SP - WT CD45.1:WT RANKL vs WT CD45.1: LTα-/- RANKL | 0.0013 | ** | T-test |
| Figure 6H | Thymic ETPs – UT vs WT CD45.1:WT PBS | 0.0346 | * | T-test |

| | | | | |
|---|---|---|---|---|
| | Thymic ETPs - WT CD45.1:WT GST vs WT CD45.1:WT RANKL | 0.0185 | * | T-test |
| | BM prethymic progenitors - UT vs WT CD45.1:WT PBS | 0.0003 | *** | T-test |
| Figure 7A | Total TECs; WT CD45.1:WT GST vs WT CD45.1:WT RANKL | 0.0476 | * | Mann Whitney test, one tailed |
| | cTECs; WT CD45.1:WT GST vs WT CD45.1:WT RANKL | 0.0476 | * | Mann Whitney test, one tailed |
| | mTECs; WT CD45.1:WT GST vs WT CD45.1:WT RANKL | 0.0476 | * | Mann Whitney test, one tailed |
| Figure 7B | WT CD45.1:WT GST vs WT CD45.1:WT RANKL | 0.0476 | * | Mann Whitney test, one tailed |
| Figure 7C | CD45.1 total donor cells; WT CD45.1:WT GST vs WT CD45.1:WT RANKL | 0.0262 | * | Mann Whitney test, one tailed |
| | DN; WT CD45.1:WT GST vs WT CD45.1:WT RANKL | 0.0476 | * | Mann Whitney test, one tailed |
| | DP; WT CD45.1:WT GST vs WT CD45.1:WT RANKL | 0.0476 | * | Mann Whitney test, one tailed |
| | CD4+ SP; WT CD45.1:WT GST vs WT CD45.1:WT RANKL | 0.0476 | * | Mann Whitney test, one tailed |
| | CD8+ SP; WT CD45.1:WT GST vs WT CD45.1:WT RANKL | 0.0476 | * | Mann Whitney test, one tailed |
| Figure 7D | ETPs; WT CD45.1:WT GST vs WT CD45.1:WT RANKL | 0.0476 | * | Mann Whitney test, one tailed |
| | | | | |
| EV1A | GST vs RANKL | 0.0143 | * | Mann Whitney test, two tailed |
| | RANKL vs RANK-Fc | 0.0143 | * | Mann Whitney test, two tailed |
| EV1B | LTα protein expression in hematopoietic cells after RANKL *in vitro* stimulation | <0.0001 | **** | One-Way ANOVA |
| EV2A | cTECs; WT vs LTα-/- | 0.0175 | * | Mann Whitney test, two tailed |
| | mTECs; WT vs LTα-/- | 0.0012 | ** | Mann Whitney test, two tailed |
| EV2B | CD80loAire- mTECs; WT vs LTα-/- | 0.0221 | * | Mann Whitney test, two tailed |
| | CD80hiAire- mTECs; WT vs LTα-/- | 0.0012 | ** | Mann Whitney test, two tailed |
| | CD80hiAire+ mTECs; WT vs LTα-/- | 0.0041 | ** | Mann Whitney test, two tailed |
| EV2C | WT vs LTα-/- | 0.0004 | *** | T-test |
| EV3A | CD4+; d21; WT CD45.1:WT vs WT CD45.1: LTα-/- | 0.0286 | * | Mann Whitney test, one tailed |
| | CD4+; d65; WT CD45.1:WT vs WT CD45.1: LTα-/- | 0.0159 | * | Mann Whitney test, one tailed |
| | CD4+; d100; WT CD45.1:WT vs WT CD45.1: LTα-/- | 0.0317 | * | Mann Whitney test, one tailed |
| | CD8+; d21; WT CD45.1:WT vs WT CD45.1: LTα-/- | 0.0143 | * | Mann Whitney test, one tailed |
| | CD8+; d65; WT CD45.1:WT vs WT CD45.1: LTα-/- | 0.0159 | * | Mann Whitney test, one tailed |
| | CD8+; d100; WT CD45.1:WT vs WT CD45.1: LTα-/- | 0.0159 | * | Mann Whitney test, one tailed |
| EV3B | Foxp3+ Tregs; d21; WT CD45.1:WT vs WT CD45.1: LTα-/- | 0.0143 | * | Mann Whitney test, one tailed |
| | Foxp3+ Tregs; d65; WT CD45.1:WT vs WT CD45.1: LTα-/- | 0.0079 | ** | Mann Whitney test, one tailed |
| | Foxp3+ Tregs; d100; WT CD45.1:WT vs WT CD45.1: LTα-/- | 0.0079 | ** | Mann Whitney test, one tailed |
| EV3C | CD4+; d21; WT CD45.1:WT vs WT CD45.1: LTα-/- | 0.0286 | * | Mann Whitney test, one tailed |
| | CD4+; d65; WT CD45.1:WT vs WT CD45.1: LTα-/- | 0.0079 | ** | Mann Whitney test, one tailed |
| | CD4+; d100; WT CD45.1:WT vs WT CD45.1: LTα-/- | 0.0079 | ** | Mann Whitney test, one tailed |
| | CD8+; d21; WT CD45.1:WT vs WT CD45.1: LTα-/- | 0.0286 | * | Mann Whitney test, one tailed |
| | CD8+; d65; WT CD45.1:WT vs WT CD45.1: LTα-/- | 0.0079 | ** | Mann Whitney test, one tail |
| | CD8+; d100; WT CD45.1:WT vs WT CD45.1: LTα-/- | 0.0286 | * | Mann Whitney test, one tailed |

| | | | | |
|---|---|---|---|---|
| EV3D | Foxp3+ Tregs; d21; WT CD45.1:WT vs WT CD45.1: LTα-/- | 0.0500 | * | Mann Whitney test, one tailed |
| | Foxp3+ Tregs; d65; WT CD45.1:WT vs WT CD45.1: LTα-/- | 0.0079 | ** | Mann Whitney test, one tailed |
| | Foxp3+ Tregs; d100; WT CD45.1:WT vs WT CD45.1: LTα-/- | 0.0079 | ** | Mann Whitney test, one tailed |
| EV3E | CD4+; WT CD45.1:WT vs WT CD45.1: LTα-/- | 0.0500 | * | Mann Whitney test, one tailed |
| | CD8+; WT CD45.1:WT vs WT CD45.1: LTα-/- | 0.0500 | * | Mann Whitney test, one tailed |
| EV3F | CD4+; WT CD45.1:WT vs WT CD45.1: LTα-/- | 0.0028 | ** | Mann Whitney test, one tailed |
| | CD8+; WT CD45.1:WT vs WT CD45.1: LTα-/- | 0.0079 | ** | Mann Whitney test, one tailed |
| EV4 | CD45.1 total donor cells; d21; WT CD45.1:WT GST vs WT CD45.1:WT RANKL | 0.0006 | *** | Mann Whitney test, one tailed |
| | CD45.1 total donor cells; d21; WT CD45.1:WT RANKL vs WT CD45.1: LTα-/- RANKL | 0.0006 | *** | Mann Whitney test, one tailed |
| | CD45.1 total donor cells; d65; WT CD45.1:WT GST vs WT CD45.1:WT RANKL | 0.0500 | * | Mann Whitney test, one tailed |
| | CD45.1 total donor cells; d65; WT CD45.1:WT RANKL vs WT CD45.1: LTα-/- RANKL | 0.0500 | * | Mann Whitney test, one tailed |
| | CD8+ T cells; d21; WT CD45.1:WT GST vs WT CD45.1:WT RANKL | 0.0002 | *** | Mann Whitney test, one tailed |
| | CD8+ T cells; d21; WT CD45.1:WT RANKL vs WT CD45.1: LTα-/- RANKL | 0.0119 | * | Mann Whitney test, one tailed |
| | CD8+ T cells; d65; WT CD45.1:WT GST vs WT CD45.1:WT RANKL | 0.0500 | * | Mann Whitney test, one tailed |
| | CD8+ T cells; d65; WT CD45.1:WT RANKL vs WT CD45.1: LTα-/- RANKL | 0.0500 | * | Mann Whitney test, one tailed |
| | CD4+ T cells; d21; WT CD45.1:WT GST vs WT CD45.1:WT RANKL | 0.0095 | ** | Mann Whitney test, one tailed |
| | CD4+ T cells; d21; WT CD45.1:WT RANKL vs WT CD45.1: LTα-/- RANKL | 0.0179 | * | Mann Whitney test, one tailed |
| | CD4+ T cells; d65; WT CD45.1:WT GST vs WT CD45.1:WT RANKL | 0.0500 | * | Mann Whitney test, one tailed |
| | CD4+ T cells; d65; WT CD45.1:WT RANKL vs WT CD45.1: LTα-/- RANKL | 0.0500 | * | Mann Whitney test, one tailed |
| | Foxp3+ Tregs; d21; WT CD45.1:WT GST vs WT CD45.1:WT RANKL | 0.0213 | * | Mann Whitney test, one tailed |
| | Foxp3+ Tregs; d21; WT CD45.1:WT RANKL vs WT CD45.1: LTα-/- RANKL | 0.0213 | * | Mann Whitney test, one tailed |
| | Foxp3+ Tregs; d65; WT CD45.1:WT GST vs WT CD45.1:WT RANKL | 0.0500 | * | Mann Whitney test, one tailed |
| | Foxp3+ Tregs; d65; WT CD45.1:WT RANKL vs WT CD45.1: LTα-/- RANKL | 0.0500 | * | Mann Whitney test, one tailed |
| EV5B | UT vs d3 SL-TBI | 0.0500 | * | Mann Whitney test, one tailed |
| EV5C | Total TECs; WT CD45.1:WT vs WT CD45.1: LTα-/- | 0.0022 | ** | Mann Whitney test, one tailed |
| | cTECs; WT CD45.1:WT vs WT CD45.1: LTα-/- | 0.0152 | * | Mann Whitney test, one tailed |
| | mTECs; WT CD45.1:WT vs WT CD45.1: LTα-/- | 0.0022 | ** | Mann Whitney test, one tailed |

| | | | | |
|---|---|---|---|---|
| EV5D | Total TECs; WT CD45.1:WT vs WT CD45.1: LTα-/- | 0.0011 | ** | Mann Whitney test, one tailed |
| | cTECs; WT CD45.1:WT vs WT CD45.1: LTα-/- | 0.0500 | * | Mann Whitney test, one tailed |
| | mTECs; WT CD45.1:WT vs WT CD45.1: LTα-/- | 0.0011 | ** | Mann Whitney test, one tailed |
| EV5E | CD80loAire- mTECs; WT CD45.1:WT vs WT CD45.1: LTα-/- | 0.0048 | ** | Mann Whitney test, one tailed |
| | CD80hiAire- mTECs; WT CD45.1:WT vs WT CD45.1: LTα-/- | 0.0152 | * | Mann Whitney test, one tailed |
| | CD80hiAire+ mTECs; WT CD45.1:WT vs WT CD45.1: LTα-/- | 0.0130 | * | Mann Whitney test, one tailed |
| EV5F | CD45.1 total donor cells; WT CD45.1:WT vs WT CD45.1: LTα-/- | 0.0043 | ** | Mann Whitney test, one tailed |
| | DN; WT CD45.1:WT vs WT CD45.1: LTα-/- | 0.0500 | * | Mann Whitney test, one tailed |
| | DP; WT CD45.1:WT vs WT CD45.1: LTα-/- | 0.0500 | * | Mann Whitney test, one tailed |
| | CD4+ SP; WT CD45.1:WT vs WT CD45.1: LTα-/- | 0.0278 | * | Mann Whitney test, one tailed |
| | CD8+ SP; WT CD45.1:WT vs WT CD45.1: LTα-/- | 0.0011 | ** | Mann Whitney test, one tailed |
| EV5G | ETPs; WT CD45.1:WT vs WT CD45.1: LTα-/- | 0.0500 | * | Mann Whitney test, one tailed |
| EV5H | CD4+ T cells; WT CD45.1:WT vs WT CD45.1: LTα-/- | 0.0152 | * | Mann Whitney test, one tailed |
| EV5I | Foxp3+ Tregs; WT CD45.1:WT vs WT CD45.1: LTα-/- | 0.0043 | ** | Mann Whitney test, one tailed |
| EV5J | CD4+ T cells; WT CD45.1:WT vs WT CD45.1: LTα-/- | 0.0119 | * | Mann Whitney test, one tailed |
| | CD8+ T cells; WT CD45.1:WT vs WT CD45.1: LTα-/- | 0.0130 | * | Mann Whitney test, one tailed |
| EV5K | Foxp3+ Tregs; WT CD45.1:WT vs WT CD45.1: LTα-/- | 0.0119 | * | Mann Whitney test, one tailed |
| | | | | |
| Appendix Figure S3 | WT CD45.1:WT vs WT CD45.1: LTα-/- | 0.0227 | * | T-test |
| Appendix Figure S4B | Total TECs; WT CD45.1:WT GST vs WT CD45.1:WT RANKL | 0.0500 | * | Mann Whitney test, one tailed |
| | Total TECs; WT CD45.1:WT RANKL vs WT CD45.1: LTα-/- RANKL | 0.0500 | * | Mann Whitney test, one tailed |
| | cTECs; WT CD45.1:WT GST vs WT CD45.1:WT RANKL | 0.0500 | * | Mann Whitney test, one tailed |
| | cTECs; WT CD45.1:WT RANKL vs WT CD45.1: LTα-/- RANKL | 0.0500 | * | Mann Whitney test, one tailed |
| | cTECs; WT CD45.1: LTα-/- GST vs WT CD45.1: LTα-/- RANKL | 0.0500 | * | Mann Whitney test, one tailed |
| | mTECs; WT CD45.1:WT GST vs WT CD45.1:WT RANKL | 0.0500 | * | Mann Whitney test, one tailed |
| | mTECs; WT CD45.1:WT RANKL vs WT CD45.1: LTα-/- RANKL | 0.0500 | * | Mann Whitney test, one tailed |
| Appendix Figure S4C | CD45.1 total donor cells; WT CD45.1:WT GST vs WT CD45.1:WT RANKL | 0.0500 | * | Mann Whitney test, one tailed |
| | CD45.1 total donor cells; WT CD45.1:WT RANKL vs WT CD45.1: LTα-/- RANKL | 0.0500 | * | Mann Whitney test, one tailed |
| | Thymic ETPs; WT CD45.1:WT GST vs WT CD45.1:WT RANKL | 0.0500 | * | Mann Whitney test, one tailed |
| | Thymic ETPs; WT CD45.1:WT RANKL vs WT CD45.1: LTα-/- RANKL | 0.0500 | * | Mann Whitney test, one tailed |
| | DN; WT CD45.1:WT GST vs WT CD45.1:WT RANKL | 0.0500 | * | Mann Whitney test, one tailed |
| | DN; WT CD45.1:WT RANKL vs WT CD45.1: LTα-/- RANKL | 0.0463 | * | Mann Whitney test, one tailed |
| | DP; WT CD45.1:WT GST vs WT CD45.1:WT RANKL | 0.0500 | * | Mann Whitney test, one tailed |

| | | | | |
|---|---|---|---|---|
| | DP; WT CD45.1:WT RANKL vs WT CD45.1: LTα-/- RANKL | 0.0500 | * | Mann Whitney test, one tailed |
| | CD4+ SP; WT CD45.1:WT GST vs WT CD45.1:WT RANKL | 0.0500 | * | Mann Whitney test, one tailed |
| | CD4+ SP; WT CD45.1:WT RANKL vs WT CD45.1: LTα-/- RANKL | 0.0500 | * | Mann Whitney test, one tailed |
| | CD8+ SP; WT CD45.1:WT RANKL vs WT CD45.1: LTα-/- RANKL | 0.0500 | * | Mann Whitney test, one tailed |
| Appendix Figure S5 | Frequency; WT CD45.1:WT GST vs WT CD45.1:WT RANKL | 0.0500 | * | Mann Whitney test, two tailed |
| | Frequency; WT CD45.1:WT RANKL vs WT CD45.1: LTα-/- RANKL | 0.0500 | * | Mann Whitney test, two tailed |
| | Numbers; WT CD45.1:WT GST vs WT CD45.1:WT RANKL | 0.0500 | * | Mann Whitney test, two tailed |
| | Numbers; WT CD45.1:WT RANKL vs WT CD45.1: LTα-/- RANKL | 0.0500 | * | Mann Whitney test, two tailed |
| Appendix Figure S6A | CD45.1 total donor cells ; PBS vs RANKL | 0.0011 | ** | Mann Whitney test, one tailed |
| | CD45.1 total donor cells ; PBS vs IL-22 | 0.0011 | ** | Mann Whitney test, one tailed |
| | CD45.1 total donor cells ; IL-22 vs IL-22 + RANKL | 0.0043 | ** | Mann Whitney test, two tailed |
| | CD45.1 total donor cells ; RANKL vs IL-22 + RANKL | 0.0043 | ** | Mann Whitney test, two tailed |
| | DN ; PBS vs IL-22 | 0.0500 | * | Mann Whitney test, one tailed |
| | DN ; PBS vs RANKL | 0.0500 | * | Mann Whitney test, one tailed |
| | DP ; PBS vs IL-22 | 0.0011 | ** | Mann Whitney test, one tailed |
| | DP ; PBS vs RANKL | 0.0011 | ** | Mann Whitney test, one tailed |
| | DP ; IL-22 vs IL-22 + RANKL | 0.0043 | ** | Mann Whitney test, two tailed |
| | DP ; RANKL vs IL-22 + RANKL | 0.0043 | ** | Mann Whitney test, two tailed |
| | CD4+SP ; PBS vs IL-22 | 0.0011 | ** | Mann Whitney test, one tailed |
| | CD4+SP ; PBS vs RANKL | 0.0011 | ** | Mann Whitney test, one tailed |
| | CD4+SP ; IL-22 vs IL-22 + RANKL | 0.0043 | ** | Mann Whitney test, two tailed |
| | CD4+SP ; RANKL vs IL-22 + RANKL | 0.0043 | ** | Mann Whitney test, two tailed |
| | CD8+ SP ; PBS vs IL-22 | 0.0011 | ** | Mann Whitney test, one tailed |
| | CD8+ SP ; PBS vs RANKL | 0.0011 | ** | Mann Whitney test, one tailed |
| | CD8+ SP ; IL-22 vs IL-22 + RANKL | 0.0011 | ** | Mann Whitney test, two tailed |
| | CD8+ SP ; RANKL vs IL-22 + RANKL | 0.0043 | ** | Mann Whitney test, two tailed |
| Appendix Figure S6B | ETPs ; PBS vs IL-22 | 0.0011 | ** | Mann Whitney test, one tailed |
| | ETPs ; PBS vs RANKL | 0.0011 | ** | Mann Whitney test, one tailed |
| | ETPs ; IL-22 vs IL-22 + RANKL | 0.0500 | * | Mann Whitney test, two tailed |
| | ETPs ; RANKL vs IL-22 + RANKL | 0.0500 | * | Mann Whitney test, two tailed |
| Appendix Figure S6C | CD45.1 total donor cells ; PBS vs IL-22 | 0.0011 | ** | Mann Whitney test, one tailed |
| | CD45.1 total donor cells ; PBS vs RANKL | 0.0011 | ** | Mann Whitney test, one tailed |
| | CD45.1 total donor cells ; IL-22 vs RANKL | 0.0022 | ** | Mann Whitney test, two tailed |
| | CD45.1 total donor cells ; RANKL vs IL-22 + RANKL | 0.0022 | ** | Mann Whitney test, two tailed |
| | CD45.1 total donor cells ; PBS vs IL-22 + RANKL | 0.0022 | ** | Mann Whitney test, two tailed |
| | CD4+ T cells ; PBS vs IL-22 | 0.0325 | * | Mann Whitney test, one tailed |
| | CD4+ T cells ; PBS vs RANKL | 0.0160 | * | Mann Whitney test, one tailed |
| | CD4+ T cells ; PBS vs IL-22 + RANKL | 0.0303 | * | Mann Whitney test, one tailed |
| | CD8+ T cells ; PBS vs IL-22 | 0.0500 | * | Mann Whitney test, one tailed |

| | | | | |
|---|---|---|---|---|
| | CD8+ T cells ; PBS vs RANKL | 0.0500 | * | Mann Whitney test, one tailed |
| | Foxp3+ Tregs ; PBS vs IL-22 | 0.0130 | * | Mann Whitney test, one tailed |
| | Foxp3+ Tregs ; PBS vs RANKL | 0.0011 | ** | Mann Whitney test, one tailed |
| | Foxp3+ Tregs ; PBS vs IL-22 + RANKL | 0.0152 | * | Mann Whitney test, two tailed |
| Appendix Figure S6D | Total TECs; PBS vs RANKL | 0.0022 | ** | Mann Whitney test, two tailed |
| | Total TECs; GST vs RANKL | 0.0500 | * | Mann Whitney test, two tailed |
| | Total TECs; RANKL vs IL-22 + RANKL | 0.0022 | ** | Mann Whitney test, two tailed |
| | cTECs; PBS vs RANKL | 0.0022 | ** | Mann Whitney test, one tailed |
| | cTECs; IL-22 vs RANKL | 0.0411 | * | Mann Whitney test, two tailed |
| | mTECs; PBS vs RANKL | 0.0022 | ** | Mann Whitney test, one tailed |
| | mTECs; IL-22 vs RANKL | 0.0500 | * | Mann Whitney test, two tailed |
| | mTECs; RANKL vs IL-22 + RANKL | 0.0022 | ** | Mann Whitney test, two tailed |
| Appendix Figure S6E | CD80loAire- ; PBS vs RANKL | 0.0411 | * | Mann Whitney test, one tailed |
| | CD80loAire- ; IL-22 vs RANKL | 0.0500 | * | Mann Whitney test, two tailed |
| | CD80loAire- ; RANKL vs IL-22 + RANKL | 0.0087 | ** | Mann Whitney test, two tailed |
| | CD80hiAire- ; PBS vs IL-22 | 0.0130 | * | Mann Whitney test, one tailed |
| | CD80hiAire- ; PBS vs RANKL | 0.0011 | ** | Mann Whitney test, one tailed |
| | CD80hiAire- ; RANKL vs IL-22 + RANKL | 0.0043 | ** | Mann Whitney test, two tailed |
| | CD80hiAire+ ; PBS vs IL-22 | 0.0130 | * | Mann Whitney test, one tailed |
| | CD80hiAire+ ; PBS vs RANKL | 0.0011 | ** | Mann Whitney test, one tailed |
| | CD80hiAire+ ; RANKL vs IL-22 + RANKL | 0.0043 | ** | Mann Whitney test, two tailed |
| Appendix Figure S6F | cTEChi ; PBS vs RANKL | 0.0022 | ** | Mann Whitney test, one tailed |
| | cTEChi ; IL-22 vs RANKL | 0.0022 | ** | Mann Whitney test, two tailed |
| | cTEChi ; RANKL vs IL-22 + RANKL | 0.0043 | ** | Mann Whitney test, two tailed |
| | mTEChi ; PBS vs IL-22 | 0.0011 | ** | Mann Whitney test, one tailed |
| | mTEChi ; PBS vs RANKL | 0.0011 | ** | Mann Whitney test, one tailed |
| | mTEChi ; RANKL vs IL-22 + RANKL | 0.0022 | ** | Mann Whitney test, two tailed |
| | mTEChi ; IL-22 vs IL-22 + RANKL | 0.0087 | ** | Mann Whitney test, two tailed |
| | TEClo ; PBS vs RANKL | 0.0022 | ** | Mann Whitney test, one tailed |
| | TEClo ; IL-22 vs RANKL | 0.0043 | ** | Mann Whitney test, two tailed |
| | TEClo ; IL-22 vs IL-22 + RANKL | 0.0022 | ** | Mann Whitney test, two tailed |
| | mTEClo ; PBS vs RANKL | 0.0043 | ** | Mann Whitney test, one tailed |
| | mTEClo ; IL-22 vs RANKL | 0.0043 | ** | Mann Whitney test, two tailed |
| | mTEClo ; IL-22 vs IL-22 + RANKL | 0.0022 | ** | Mann Whitney test, two tailed |
| Appendix Figure S6G | PBS vs RANKL | 0.0043 | ** | Mann Whitney test, one tailed |
| | IL-22 vs RANKL | 0.0087 | ** | Mann Whitney test, two tailed |
| | RANKL vs IL-22 + RANKL | 0.0022 | ** | Mann Whitney test, two tailed |
| Appendix Figure S7A | CD4+ T cells; WT CD45.1:WT GST vs WT CD45.1:WT RANKL | 0.0476 | * | Mann Whitney test, one tailed |
| | CD8+ T cells; WT CD45.1:WT GST vs WT CD45.1:WT RANKL | 0.0476 | * | Mann Whitney test, one tailed |
| Appendix Figure S7B | Foxp3+ Tregs; WT CD45.1:WT GST vs WT CD45.1:WT RANKL | 0.0476 | * | Mann Whitney test, one tailed |
| Appendix Figure S9A | Area; GST vs RANKL-GST | <0.0001 | **** | T-test |

| | | | | |
|---|---|---|---|---|
| Appendix Figure S9B | Frequency; GST vs RANKL-GST | 0.0043 | ** | Mann Whitney test, two tailed |
| | Numbers; GST vs RANKL-GST | 0.0043 | ** | Mann Whitney test, two tailed |
