# Supplementary material for: Administration of RANKL boosts thymic regeneration upon bone marrow transplantation
Source: EMBO Mol Med. 2017 Apr 28;9(6):835–51. doi: 10.15252/emmm.201607176 (PMC5452038; doi:10.15252/emmm.201607176)
Supplement: Supplementary file 2 — Expanded View Figures PDF [file EMMM-9-835-s002.pdf]

# Expanded View Figures

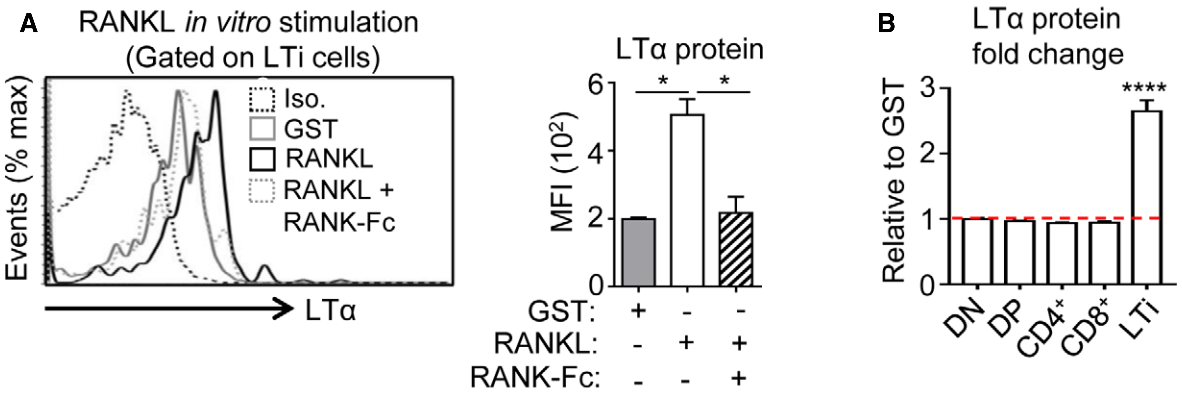


**Figure EV1. *In vitro* stimulation with RANKL induces the upregulation of LTα specifically in thymic LTi cells.**

A LTα protein expression was analyzed by flow cytometry in thymic LTi cells from sublethally irradiated WT mice treated *in vitro* for 24 h with GST, RANKL-GST, or RANKL-GST + RANK-Fc. The histogram shows the MFI of LTα for each condition. Iso: Isotype control.

B LTα protein was analyzed in DN, DP, $CD4^+$, and $CD8^+$ SP as well as in LTi cells purified from sublethally irradiated WT mice and treated *in vitro* for 24 h with GST or RANKL-GST. Results are represented as fold change relative to the GST condition.

Data information: Data are shown as mean ± SEM and are pooled of two independent experiments with similar results ($n$ = 3 mice per group). *$P < 0.05$; ****$P < 0.0001$. Exact $P$-values and statistical tests used to calculate them are provided in Appendix Table S2.

 © 2017 The Authors

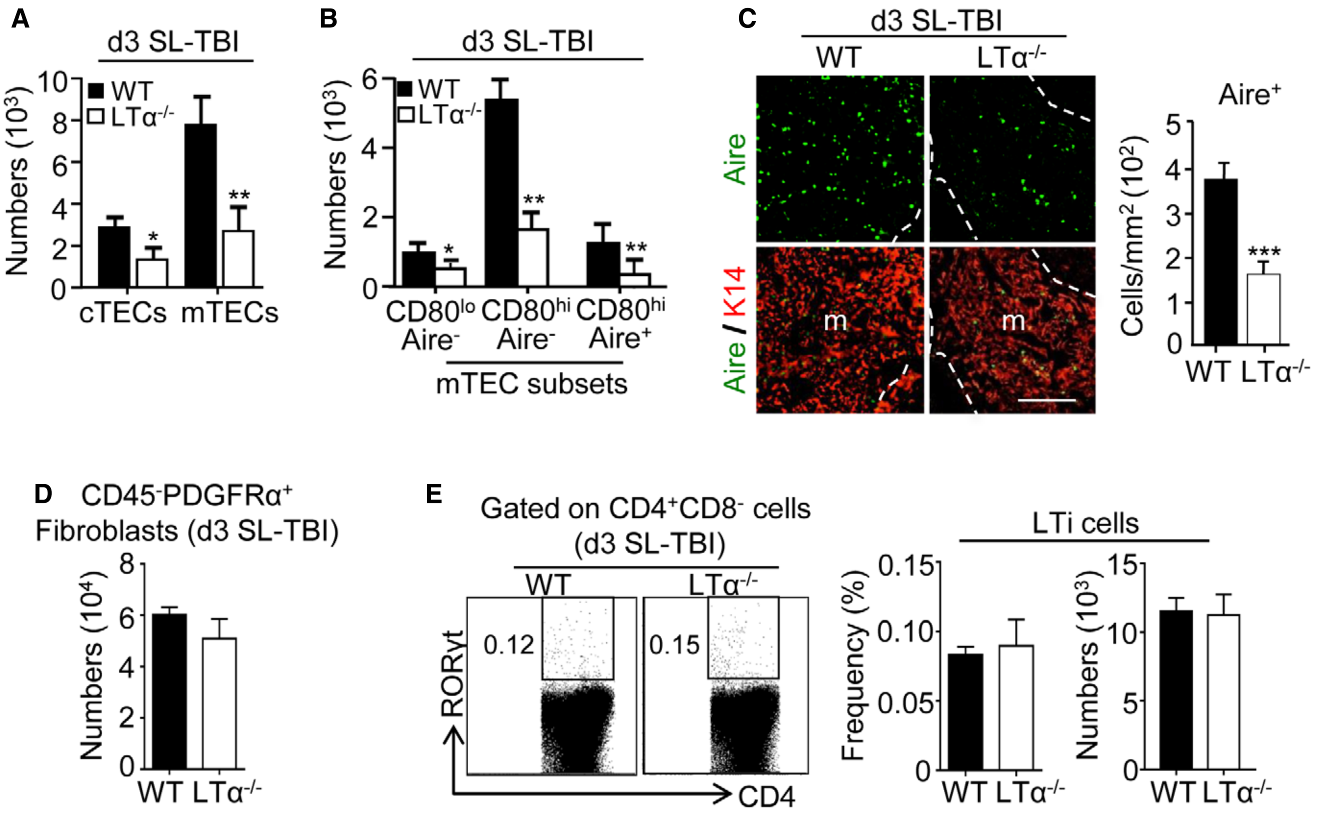


**Figure EV2. TECs but not fibroblasts and LTi cells are severely reduced in LTα$^{-/-}$ mice at d3 SL-TBI.**

A, B Histograms show numbers of cTECs and mTECs (A) as well as mTEC subsets (B) in WT and LTα$^{-/-}$ mice at d3 SL-TBI.

C Thymic sections from WT and LTα$^{-/-}$ mice at d3 SL-TBI were stained for the expression of K14 and Aire. The histogram shows the density of Aire$^{+}$ cells in medullary area. m, medulla. Fifteen sections were quantified; scale bar: 100 μm.

D The histogram shows numbers of CD45$^{-}$PDFRα$^{+}$ fibroblasts in WT and LTα$^{-/-}$ mice at d3 SL-TBI.

E Flow cytometry profiles and frequencies of thymic LTi cells from WT or LTα$^{-/-}$ mice at d3 SL-TBI.

Data information: Data are shown as mean ± SEM and are pooled of five independent experiments with similar results ($n$ = 3 mice per group). *$P$ < 0.05; **$P$ < 0.01; ***$P$ < 0.001. Exact $P$-values and statistical tests used to calculate them are provided in Appendix Table S2.

© 2017 The Authors 

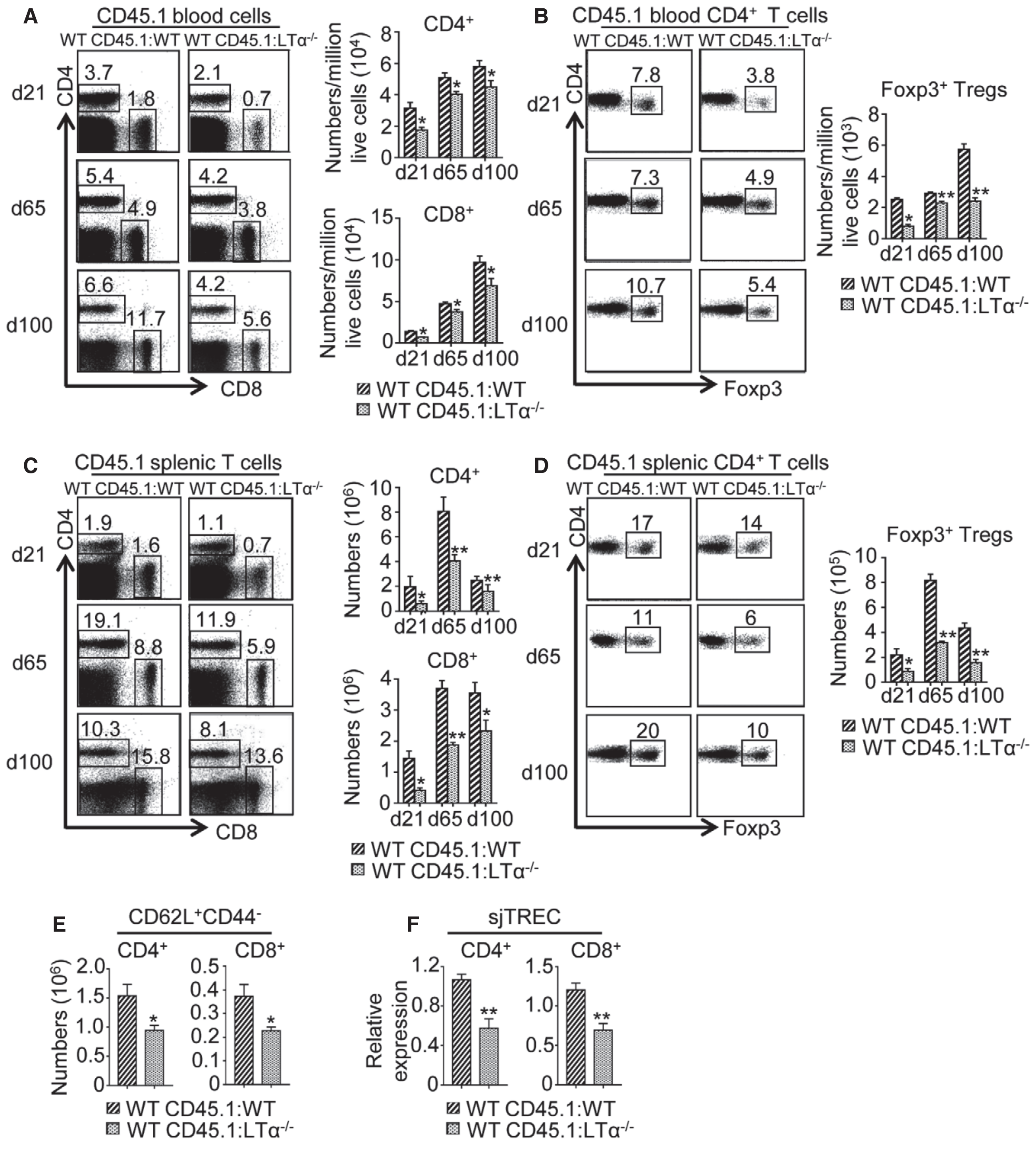


**Figure EV3. LTα expression during BMT is required for peripheral T-cell reconstitution.**

A–D Flow cytometry profiles and numbers of $CD4^+$ and $CD8^+$ T cells (A, C) as well as $CD4^+Foxp3^+$ Tregs (B, D) from CD45.1 donor origin in blood (A, B) and spleen (C, D) of WT CD45.1:WT and WT CD45.1:LTα$^{-/-}$ mice at d21, d65, and d100 upon BMT. Significance relative to WT CD45.1:WT chimeras.

E Histograms show numbers of $CD62L^+CD44^-$ naïve $CD4^+$ and $CD8^+$ T cells in the spleen of WT CD45.1:WT and WT CD45.1:LTα$^{-/-}$ mice at d21 pBMT.

F sjTREC were quantified by qPCR from genomic DNA of cell-sorted splenic $CD4^+$ and $CD8^+$ T cells from WT CD45.1:WT and WT CD45.1:LTα$^{-/-}$ mice at d21 pBMT.

Data information: Data are shown as mean ± SEM and are pooled of two independent experiments with similar results ($n$ = 3–5 mice per group). *$P < 0.05$; **$P < 0.01$; one-tailed Mann–Whitney $U$-test. Exact $P$-values are provided in Appendix Table S2.

 © 2017 The Authors

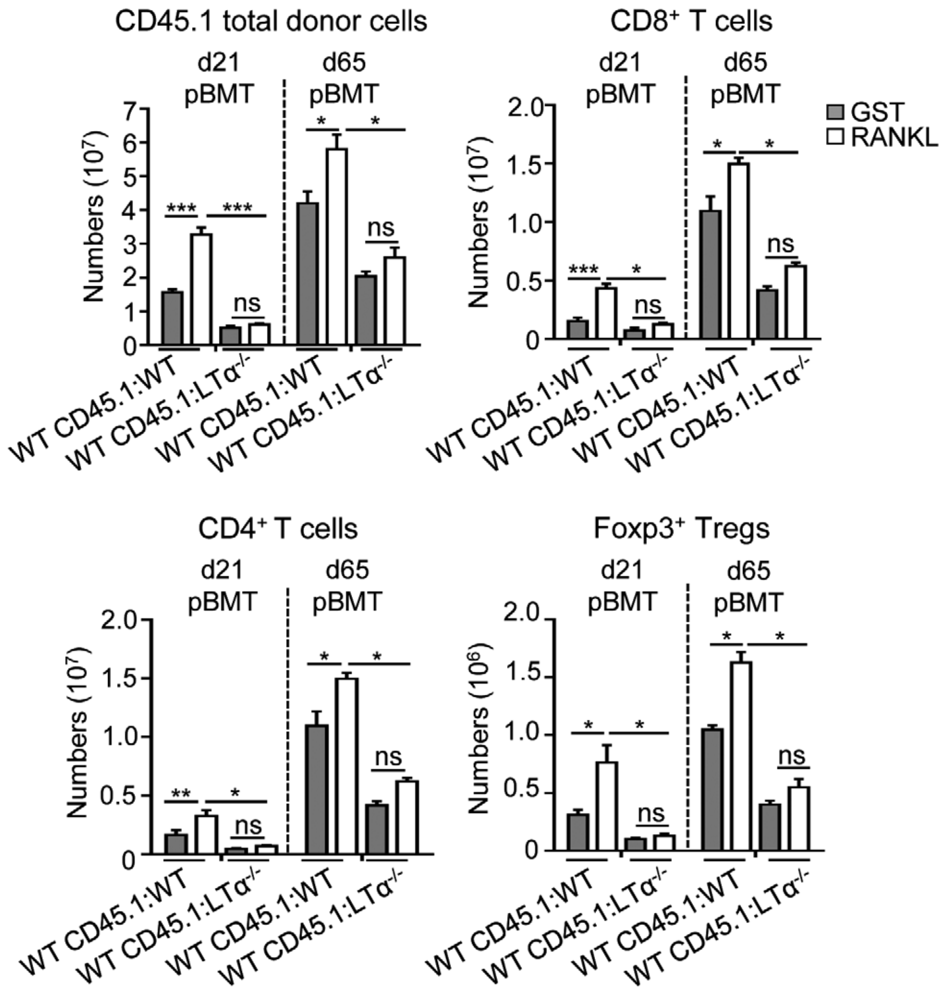


**Figure EV4. RANKL treatment early after BMT boosts peripheral T-cell reconstitution optimally in an LTα-dependent manner.**

Histograms show numbers of total cells and $CD4^+$ and $CD8^+$ T cells as well as $CD4^+Foxp3^+$ Tregs from CD45.1 donor origin in the spleen of WT CD45.1:WT and WT CD45.1:$LT\alpha^{-/-}$ mice treated with GST or RANKL proteins at d2, d4, and d6 pBMT and analyzed at d21 and d65 pBMT. pBMT: post-bone marrow transplantation. Data are shown as mean ± SEM and are pooled of three independent experiments with similar results ($n$ = 3–5 mice per group). $^*P < 0.05$; $^{**}P < 0.01$; $^{***}P < 0.001$; one-tailed Mann–Whitney $U$-test. Exact $P$-values are provided in Appendix Table S2.

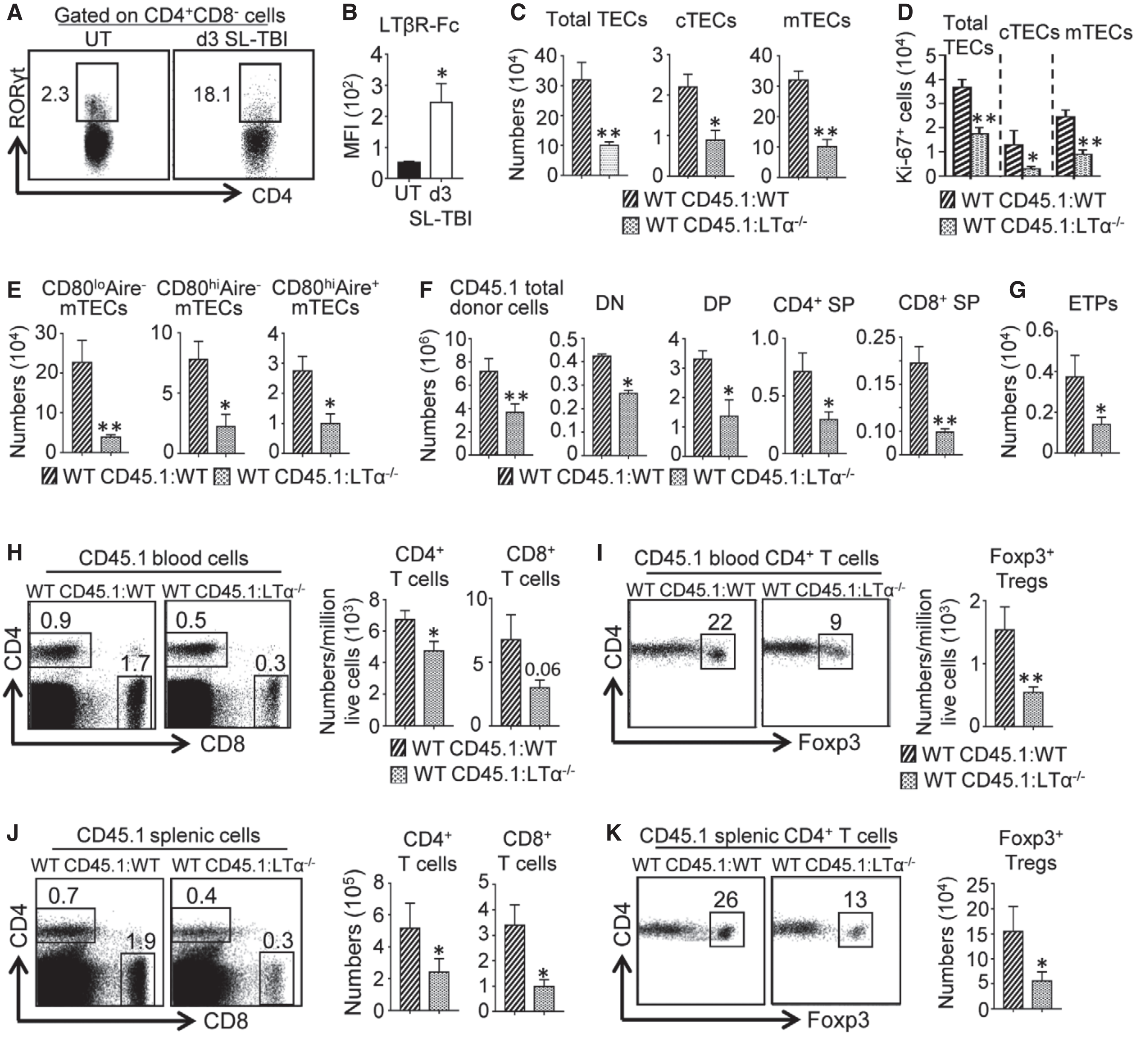


**Figure EV5. The critical role of LTα in thymic regeneration and peripheral T-cell reconstitution persists with age.**

A CD4$^+$CD8$^-$ thymic cells from 8-month-old UT WT mice ($n$ = 9) or at d3 SL-TBI ($n$ = 6) were analyzed for the expression of RORγt by flow cytometry.

B MFI of LTβR-Fc staining in thymic LTi cells from 6- to 8-month-old UT WT mice or at d3 SL-TBI.

C–G Histograms show numbers of total TECs, cTECs, mTECs (C); Ki-67$^+$ TEC subsets (D); mTEC subsets (E); total thymic cells, T-cell subsets (DN, DP, CD4$^+$ SP, and CD8$^+$ SP) (F); and ETPs (G) in the thymus from WT CD45.1:WT and WT CD45.1:LTα$^{-/-}$ chimeras of 6–8 months of age at d21 upon BMT.

H–K Flow cytometry profiles and numbers of CD4$^+$ and CD8$^+$ T cells (H, J) as well as CD4$^+$Foxp3$^+$ Tregs (I, K) from CD45.1 donor origin in blood (H, I) and spleen (J, K) of WT CD45.1:WT and WT CD45.1:LTα$^{-/-}$ mice of 6–8 months of age at d21 pBMT. Significance relative to WT CD45.1:WT chimeras.

Data information: Data are shown as mean ± SEM and are pooled of two independent experiments with similar results ($n$ = 3 mice per group). *$P < 0.05$; **$P < 0.01$; one-tailed Mann–Whitney $U$-test. Exact $P$-values are provided in Appendix Table S2.

 © 2017 The Authors
